# Exploring the Potential of Supercritical Fluid Extraction of *Matricaria chamomilla* White Ray Florets as a Source of Bioactive (Cosmetic) Ingredients

**DOI:** 10.3390/antiox12051092

**Published:** 2023-05-12

**Authors:** Laura Pastare, Marta Berga, Liene Kienkas, Martins Boroduskis, Anna Ramata-Stunda, Dace Reihmane, Maris Senkovs, Gundars Skudrins, Ilva Nakurte

**Affiliations:** 1 Institute for Environmental Solutions, “Lidlauks”, Priekuli Parish, LV-4126 Cesis, Latvia; marta.berga@vri.lv (M.B.); gundars.skudrins@vri.lv (G.S.); ilva.nakurte@vri.lv (I.N.); 2 Field and Forest, SIA, 2 Izstades Str., Priekuli Parish, LV-4126 Priekuli, Latvia; liene.kienkas@fieldandforest.lv; 3 Alternative Plants, SIA, 2 Podraga Str, LV-1023 Riga, Latvia; martins@alternativeplants.eu (M.B.); anna@alternativeplants.eu (A.R.-S.); dace@alternativeplants.eu (D.R.); 4 Faculty of Biology, University of Latvia, 1 Jelgavas Str., LV-1004 Riga, Latvia; maris.senkovs@lu.lv

**Keywords:** supercritical fluid extraction, *Matricaria chamomilla*, essential oils, flavonoids, gas chromatography, liquid chromatography, mass spectrometry, antimicrobial activity, cytotoxicity

## Abstract

Aromatic and medicinal plants are a great source of useful bioactive compounds for use in cosmetics, drugs, and dietary supplements. This study investigated the potential of using supercritical fluid extracts obtained from *Matricaria chamomilla* white ray florets, a kind of industrial herbal byproduct, as a source of bioactive cosmetic ingredients. Response surface methodology to optimize the supercritical fluid extraction process by analyzing the impact of pressure and temperature on yield and the main bioactive compound groups were used. High-throughput 96-well plate spectrophotometric methods were used to analyze the extracts for total phenols, flavonoids, tannins, and sugars, as well as their antioxidant capacity. Gas chromatography and liquid chromatography–mass spectrometry was used to determine the phytochemical composition of the extracts. The extracts were also analyzed for antimicrobial activity, cytotoxicity, phototoxicity, and melanin content. Statistical analysis was performed to establish correlations between the extracts and develop models to predict the targeted phytochemical recovery and chemical and biological activities. The results show that the extracts contained a diverse range of phytochemical classes and had cytotoxic, proliferation-reducing, and antimicrobial activities, making them potentially useful in cosmetic formulations. This study provides valuable insights for further research on the uses and mechanisms of action of these extracts.

## 1. Introduction

The German chamomile (*Matricaria chamomilla* L.), belonging to the Asteraceae family, is native to southern and eastern Europe but is also found in South and North America, Australia, Asia, and other regions. Chamomile is an annual or perennial flowering plant with flowers arranged in heads with white outer ring ray florets and yellow inner disc florets [1]. It is one of the oldest and most common medicinal plants used worldwide for various applications. It has been used in herbal tea or in other various extraction forms such as herbal remedies, cosmetics, food flavors, dyes, and pest repellants. It is used topically for skin irritation, infections, and wound healing, orally for inflammation, digestive problems, and gastrointestinal disturbances, and as a mild sedative for anxiety [1,2].

The main bioactive compounds of chamomile are coumarins (herniarin and umbelliferone), flavonoids (apigenin, luteolin, patuletin, rutin, and quercetin), terpenoids (α-bisabolol, bisabolol oxides A and B, and chamazulene), organic acids, and polysaccharides. The chemical composition of plants varies depending on the type of soil, specific environment, climatic conditions, and genotype [2,3]. German chamomile contains 0.24–1.9% essential oil; the main constituents are α-bisabolol, chamazulene, (E)-β-farnesene, and their derivatives. The essential oil is deep blue due to the presence of chamazulene. Chamazulene is an artifact degraded from proazulene and matricin naturally present in plants during the distillation process. Essential oil is known for its anti-inflammatory, antiseptic, and antiphlogistic properties [1]. Apigenin is one of the main bioactive compounds found in German chamomile, and according to the European Pharmacopoeia, dried capitula must contain a minimum of 0.25% total apigenin 7-glucoside. Apigenin has shown anti-inflammatory, antioxidant, and anticarcinogenic properties, and the potential of apigenin as a natural chemopreventive agent is continuously evaluated [4]. Anticancer studies using chamomile focus mostly on apigenin as the bioactive compound. In recent studies, growth inhibitory effects on several human cancer lines were observed for chamomile extracts. Several mechanisms of action of apigenin for cancer prevention and therapy have been identified [5]. The topical use of chamomile extracts as antiphlogistic agents has been studied. A study of human skin penetration demonstrated that chamomile flavonoids (apigenin and luteolin) were absorbed on the skin’s surface and penetrated deeper layers of the skin [6]. Another study in mice demonstrated that topical application of chamomile oil on allergic dermatitis significantly reduced histamine levels [7]. Bisabolol derived from another species of *Matricaria* has anti-inflammatory effects and the capability to alleviate atopic dermatitis [8]. Simultaneously, several compounds and their derivatives present in *M. chamomilla* have been identified as dermal sensitizers (e.g., spirolactones, herniarin) [9,10]. The components of German chamomile show promising positive effects on the mechanisms of pathogenesis of eczema [11] and atopic dermatitis [7].

Previous studies have shown that chamomile extracts [12,13], essential oils [14], flavonoids [15], and polysaccharides [16,17] exhibit the ability to eliminate 1,1-diphenyl-2-picrylhydrazyl (DPPH) and hydroxyl free radicals. These compounds possess antioxidant properties that can protect skin from damage induced by free radicals, which are unstable molecules that can harm skin cells and accelerate the ageing process. Environmental factors such as UV radiation, pollution, and other stressors produce free radicals [18]. In cosmetics, chamomile is often used in products designed for sensitive or irritated skin, as it can help soothe and calm the skin. Chamomile extracts are also used in products designed to reduce the appearance of fine lines and wrinkles, as antioxidants can help improve skin elasticity and firmness [19,20].

The cultivation and processing of medicinal and aromatic plants can be an energy- and resource- intensive process. As there has been a shift back to natural medicine, the demand for medicinal plants has been increasing, especially from the cosmetic and food industries. To optimize the processing of already-cultivated plant biomass, the valorization of plant waste material is a rising topic of interest [21]. Traditional uses of plant materials create huge amounts of agricultural waste, byproducts, and possible coproducts. These plant material streams could be a great source of bioactive compounds [22].

Research in the last decade has shown that there is an increasing interest in valorization and biorefinery approaches with respect to the handling of agricultural byproducts and waste. Alcoholic and ethyl acetate extracts of byproducts of *Crocus sativus* L. contain compounds valuable for food valorization and potentially antiaging and anticancer abilities [23]. Dynamic maceration with various solvents from the oil-exhausted biomass of *Lavandula angustifolia* Mill. and *Lavandula x intermedia* Emeric ex Loisel showed the presence of bioactive compound groups such as polyphenols (19.22 ± 4.16 and 17.06 ± 3.31 mg g^−1^, respectively) and flavonoids (1.56 ± 0.21 and 1.41 ± 0.10 mg g^−1^, respectively) [24]. Research by Slavov [25] showed that *Lavandula angustifolia* Mill. waste after steam distillation and carbon dioxide (CO_2_) extraction is a promising source of phenolic acids (2.62 ± 0.19 and 1.39 ± 0.14 mg g^−1^ dry weight, respectively) and antioxidants (DPPH 355.48 ± 23.12 and 283.21 ± 17.04 μmol TE g^−1^ dry weight, respectively), with potential application in foodstuffs. Soxhlet extraction with various solvents of *Cymbopogon winterianus* Jowitt distillation waste indicated the potential of these byproducts as a natural antioxidant source [26].

Biologically active compounds can be separated from biomass using various techniques. Conventional extraction techniques such as maceration, Soxhlet extraction, and percolation and green extraction techniques such as supercritical fluid extraction (SCFE), ultrasound extraction, and microwave extraction are used for valorization purposes. The main advantage of green or clean technologies is the reduction in energy, resources, and time used, as well as the reduced toxicity of solvents used [27]. Supercritical fluid extraction with carbon dioxide is considered green extraction because CO_2_ is nontoxic, generally recognized as safe, and easily removable from the final product. SCFE is a nonexplosive, inexpensive process, and the technology is readily available. The extraction capacity of supercritical carbon dioxide is good because of its changeable nature under supercritical conditions. It can be used to extract nonpolar compounds when used alone or more polar compounds when using polar cosolvents such as ethanol or methanol [27]. The main drawbacks of SCFE are the setup costs and the required technical knowledge, especially when working with new input materials. While these extraction techniques hold great promise, the efficacy of the extraction methods and the chemical profiles they produce can vary significantly [28,29]. 

The main objective of the study was to investigate the potential of supercritical fluid extracts from *Matricaria chamomilla* white ray florets as a sustainable source of bioactive cosmetic ingredients. 

To optimize the supercritical fluid extraction of *M. chamomilla*, the response surface methodology was applied to assess the impact of the extraction parameters (pressure and temperature) on yield and the main bioactive compound groups. Liquid chromatography–high-resolution mass spectrometry (LC-qTOF-MS) and gas chromatography mass spectrometry (GC-MS) were used to chemically characterize the extracts. High-throughput 96-well plate spectrophotometric assays were used to detect the antioxidant activity of the extracts as a measure of their capacity to scavenge stable DPPH radicals. Finally, the selected extracts were screened for their antimicrobial, cytotoxic, phototoxic, and melanogenesis-regulating activities.

## 2. Materials and Methods

### 2.1. Plant Materials

*Matricaria chamomilla* white ray florets were provided by Field and Forest, Ltd. (Priekuli, Latvia), which grows, harvests, dries, and processes organic German chamomile. During the processing of chamomile herbal biomass, white ray florets from broken flowerheads are separated as a byproduct stream. The byproduct flow contains more than 95% of white ray florets and less than 5% of yellow disc florets. The moisture content of the white ray florets was 11.24 ± 0.37%, determined with a RADWAG MA 210.X2.IC.A.WH (Radom, Poland) moisture analyzer. Prior to the extraction, white ray florets were pulverized using a universal dry product mill MLVS M500 (Mazeikiai, Lithuania). The average particle diameter was 0.5 mm.

### 2.2. Supercritical Fluid Extraction

Supercritical fluid extraction was carried out with a pilot-scale supercritical fluid extractor CAREDDI SCFE-5L (UAB Analytical Solutions; Kaunas, Lithuania) using carbon dioxide (CO_2_) and ethanol (EtOH) as cosolvent. The supercritical extraction unit consisted of a high-pressure CO_2_ pump, a cosolvent pump, a cooling system, a heating system, a temperature control system, a CO_2_ storage tank, a 5 L extraction tank, and two-stage separation tanks (4 L and 2 L). Flow rates and temperatures were automatically controlled. The pressure in the extraction tank and both separation tanks was controlled by manual back pressures. The SCFE pressure and temperature used in this study is provided in Section 2.3. Separation pressure of 5 MPa was used. Dynamic extraction with a CO_2_ flow of 100 L h^−1^ and ethanol flow of 4 mL min^−1^ was used. The sample size was 600 g of pulverized white ray florets, and an extraction duration of 2 h was used.

After extraction, the extract obtained was placed in dark glass vials, sealed, and stored at 4 °C to prevent possible degradation. The extraction yield was determined gravimetrically and expressed as mL extract per 100 g^−1^ dry weight (DW) of white ray florets.

### 2.3. Response Surface Methodology 

One of the objectives of this work is to evaluate the influence of pressure and temperature on the SCFE process and optimize the conditions to obtain the best yield. To statistically study this, the response surface methodology (RSM) based on Central Composite Design (CCD), Face Centered, was used. The extraction pressure values were set between 5 and 25 MPa and the extraction temperature values between 20 °C and 55 °C, with 3 factor levels (see Table 1). The rest of the parameters (duration, CO_2_ flow rate, and cosolvent flow rate) in the experiments were constant and were based on previous experience (see Section 2.2). This design provides 13 random experiments, 5 of which are replicates of central conditions. The software used to perform the RSM with CCD Face Centered was Develve^®^ Version 4.14.1.0 (Develve, Velp, The Netherland). This software was also used for the statistical analysis of RSM.

RSM is also applied to determine the influence of extraction temperature and pressure on secondary metabolite groups and the antioxidant activity described in Section 2.6.

### 2.4. Chemicals and Reagents

The CO_2_ used in SCFE was food-grade (purity > 99.8%) and purchased from Elmemesser (Riga, Latvia). Ethanol (96%) used in the SCFE was purchased from Kalsnavas Elevators Ltd. (Jaunkalsnava, Latvia).

LC-MS-grade acetonitrile, methanol, and formic acid were purchased from Fisher Scientific (Loughborough, UK), and the water for the LC-qTOF-MS analysis was purified using a Smart2Pure water purification system (Thermo Scientific, Braunschweig, Germany). Gallic acid and aluminum chloride (AlCl_3_), cyclohexane, and trolox were purchased from Acros Organics (Geel, Belgium), Na_2_CO_3_ and NaNO_2_ were obtained from Honeywell (Charlotte, NC, USA), apigenin standard from Rotichrom, and Carl Roth GmbH (Karlsruhe, Germany), tannic acid, 2,2-diphenyl-1-picrylhydrazyl (DPPH), phenol, and coumarin from Alfa Aesar (Kandel, Germany). Folin–Ciocalteu reagent, H_2_SO_4_, D-glucose, ferulic acid, and NaOH reagents were purchased from Fisher Scientific (Loughborough, UK). Sigma-Aldrich (St. Louis, MO, USA) standards include leucine and α-bisabolol.

Mueller–Hinton broth was purchased from Biolife (Milan, Italy), malt extract from Oxoid (Cheshire, UK), and Wilkins–Chalgren broth from Sigma (St. Louis, MO, USA). DMEM medium was purchased from Sigma (Irvine, UK); penicillin–streptomycin as well as calf serum broth were purchased from Sigma (St. Louis, MO, USA); and phosphate-buffered saline was purchased from Sigma (Irvine, UK). Neutral Red dye and glacial acetic acid were purchased from Sigma (Irvine, UK).

### 2.5. Quantification of Essential Oils and Waxes

The quantification of essential oils and waxes in the extract was carried out according to the method described previously [30] by placing the extract on Petri plates and taking them to an oven with air circulation at 160 °C for 6 h. After that, the Petri plates were allowed to cool down in a desiccator and weighed. This procedure was repeated until the mass of the extract remained constant.

### 2.6. Phytochemical Screening of Secondary Metabolites by 96-Well Plate Spectrophotometric Assays

The analysis and phytochemical screening of secondary metabolites such as phenols, flavonoids, tannins, and sugars, as well as antiradical activity, were carried out according to standard procedures [30] that are briefly described in Table 2. The extracts were diluted to appropriate dilutions.

### 2.7. UHPLC-HRMS Analysis

In total, 100 mg of SFE sample was precisely weighed and placed into a PTFE centrifuge tube. Then, 10 mL of 96% ethanol was added precisely, followed by vortexing for 30 s at 2000 rpm. Samples were centrifuged for 10 min at 4400 rpm, and the supernatant was collected. The supernatant was then filtered through a 0.45 µm RC (regenerated cellulose) filter before being injected into a chromatographic system. Separation was performed according to the method described previously [30]. The obtained extracts were analyzed using an Agilent 1290 Infinity II series high-performance liquid chromatography (HPLC) system combined with an Agilent 6530 qTOF MS system (Agilent Technologies, Deutschland GmbH, Waldbronn, Germany). A Zorbax Eclipse Plus C18 Rapid Resolution HD (2.1 × 150 mm, 1.8 μm particle size) column was used with a flow rate of 0.3 mL/min. The column oven was set at 50 °C, and the sample injection volume was 1 μL with a 30 s needle wash (using 70% methanol). The mobile phase consisted of a combination of solvent A (0.1% formic acid in water) and solvent B (0.1% formic acid in acetonitrile). The gradient elution program was used as follows: initial 2% B, 0–2 min 2% B, 2–10 min 40% B, 10–20 min 80% B, 20–27 min 95% B, 27–40 min 95% B, and 40–42 min 1% B. The adjusted operating parameters of the mass spectrometer were set as follows: fragmentation: 70 V; gas temperature: 325 °C; drying gas flow: 10 L/min; nebulizer: 20 psi; sheath gas temperature: 400 °C; and sheath gas flow: 12 L/min. Electrospray ionization (ESI) was used as a source when operating in positive mode. Mass spectra in the *m*/*z* range of 50 to 2000 were obtained. The internal reference masses of 121.050873 m/z and 922.009798 m/z (G1969-85001 ESI-TOF Reference Mass Solution Kit, Agilent Technologies and Supelco) were used for all analyses of the samples. The Agilent MassHunter Qualitative Analysis 10.0 data acquisition software was used to analyze LCMS data, and the Agilent MassHunter METLIN Metabolomics Database and LipidMaps Database were used for the identification of isolated compounds. Targeted individual standards were prepared for individual compound and class quantification, as listed in Table 3.

### 2.8. GC-MS Analysis

In total, 10 mg of SFE sample was precisely weighed and placed into a PTFE centrifuge tube. Then, 10 mL of cyclohexane was added precisely, followed by vortexing for 30 s at 2000 rpm. Samples were centrifuged for 10 min at 4400 rpm, and the supernatant was collected. The supernatant was then filtered through a 0.45 µm RC (regenerated cellulose) filter before being injected into a chromatographic system. Separation was performed according to the method described previously [30]. Analyses were performed on an Agilent Technologies 7820A gas chromatograph coupled to Agilent 5977B mass selective detector (MSD) equipment. A nonpolar HP-5 capillary column (60 m × 0.25 mm; 0.25 µm film thickness) coated with 5% phenyl and 95% methyl polysiloxane was used for separation. The carrier gas was helium (He) with a split ratio of 1:100 and a flow rate of 1.5 mL/min. The volume of the injection was 3 μL. The temperature program started at 70 °C and was increased at a rate of 5 °C/min to 230 °C, followed by an increase to 295 °C at a rate of 7 °C/min. Finally, 295 °C was maintained for 30 min. The injector temperature was set at 270 °C. The mass spectra were recorded at 70 eV in the mass range of 70–500 *m*/*z*. The ion source temperature was maintained at 230 °C. The components were identified based on their retention indices, determined with reference to homologous series of C5–C24 n-alkanes, by comparison of their mass spectra with those stored in the NIST (National Institute of Standards and Technology, Geithersburg, MD, USA) MS Search 2.2 library. The Agilent MassHunter Qualitative Analysis 10.0 data acquisition software was used to analyze GC-MS data. The content of the separated compounds was calculated based on peak areas using the α-bisabolol calibration curve, which was calculated with five different concentrations ranging from 0.01 to 1.0 mg/mL and triplicate injections at each concentration level (R^2^ = 0.9989).

### 2.9. Cytotoxicity

Cytotoxicity was assessed according to OECD Test Guideline 129 [31]. BALB/3T3 cells (ATCC) (passages 12–16) were seeded in 96-well microplates at a density of 4 × 10^3^ cells/well. Cells were propagated in 100 μL S10 medium (DMEM medium supplemented with 1% penicillin (100 U mL^−1^)–streptomycin (100 μg mL^−1^) (P/S) and 10% calf serum (CS) and incubated overnight at 37 °C, 5% CO_2_. Cells were rinsed with phosphate-buffered saline (PBS), and 100 μL of S5 medium (DMEM medium supplemented with 1% P/S and 5% CS) and extract mix was added to the cells. Furthermore, wells with vehicle (appropriate solvent), S5 medium, and sodium dodecyl sulfate (SDS) in S5 medium controls were prepared. After 48 h incubation at 37 °C, 5% CO_2_, the cells were rinsed with PBS, and 250 μL of 25 μg mL^−1^ Neutral Red dye solution in S5 medium was added to all wells. The plate was incubated for 3 h at 37 °C, 5% CO_2_, the cells were rinsed with PBS, and 100 μL NR desorb solution (50% ethanol, 1% glacial acetic acid, 49% water) was added to all wells. The plate was covered and placed in a microplate shaker for 20–45 min, then removed 5–10 min before reading. The absorption at 540 nm was measured using a Tecan M200 Infinite Pro microplate reader (Tecan, Mannedorf, Switzerland). Cell viability was calculated as the percentage of the media control value. Curve-fit analysis was performed to calculate IC_50_ values. GraphPad Prism 9 software (GraphPad Software, Boston, Ma, USA) was used for statistical analysis.

### 2.10. Phototoxicity

The in vitro phototoxicology protocol was a modification of the procedure described in OECD Test Guideline 432 [32]. Balb/c 3T3 fibroblast cells were incubated with extracts in 96-well plates for 1 h and then exposed to UVA light (5 J cm^−2^) using UVACUBE 400 (Honle UV Technology, Gilching, Germany). In parallel, cells were exposed to the extracts in the dark and evaluated in parallel. Neutral Red dye uptake (NRU) was determined 24 h later, as described in Section 2.9.

### 2.11. Antimicrobial Activity

Antimicrobial activity was assessed according to the method of Wiegand et al. (2008) with slight modifications [33]. Mueller–Hinton broth was used for susceptibility testing using a two-fold serial broth microdilution assay of *Staphylococcus aureus* ATCC 6538P, *Pseudomonas aeruginosa* ATCC 9027, and *Escherichia coli* ATCC 25922. Malt extract broth was used for the testing of *Candida albicans* ATCC 10261.

The inoculum of microorganisms was prepared in sterile water with a density of 0.08–0.10 at 625 nm and diluted 100-fold in an appropriate broth. Then, 96-well plates were incubated at 37 °C for 24 h. The MIC was determined as the lowest concentration of the studied material, which did not show visible growth of microorganisms. From wells where growth was not detected, 4 μL of media was seeded on appropriate solidified media for MBC/MFC determination. 

### 2.12. Proliferation and Viability of Melanocytes

Proliferation and viability of melanocyte was assessed by MTT assay according to Cengiz et al., with modifications [34]. The B-16 (ATCC) melanocyte cell line was seeded in 96-well plates in 100 μL Dulbecco S10 medium (DMEM medium supplemented with 1% penicillin (100 U mL^−1^)–streptomycin (100 μg mL^−1^) (P/S) and 10% fetal bovine serum (FBS) and incubated overnight at 37 °C, 5% CO_2_. Extracts were added and incubated for another 24 h. After incubation, the media was removed, and 0.5 mg mL^−1^ MTT (3-[4,5-dimethylthiazol-2-yl]-2,5 diphenyl tetrazolium bromide) solution (Sigma, St. Louis, MO, USA) in 5% serum containing cultivation media was added to each well. Plates were incubated for 2 h at 37 °C and 5% CO_2_ to allow insoluble formazan precipitates to form as a result of the metabolic activity of viable cells. After incubation, the media was removed and 0.1 mL DMSO was added, and the plate was incubated for 10 min at room temperature with gentle shaking to dissolve the formazan precipitate. Absorption was measured at 570 nm using Tecan Infinite^®^ 200 PRO (Tecan Group Ltd., Mannedorf, Switzerland).

Blank wells without cells were used for background measurements. Proliferation relative to control was calculated using the equation below:

(1) $$Proliferation\left(\%\right)=\frac{{Abs}_{570}\left(treatment)-{Abs}_{570}(background\right)}{{Abs}_{570}\left(untreated\;control)-{Abs}_{570}(background\right)}\times 100\%.$$

Three replicates were analyzed throughout the study. Average ± standard deviation (SD) was used to express the experimental values. One-way analysis of variance (ANOVA) with Tukey’s multiple comparison test was used for statistical analysis. A *p*-value < 0.05 was considered statistically significant.

### 2.13. Quantification of Melanin Content

Changes in melanin content were evaluated in the human melanocyte cell line FM-55 (ATCC) and murine melanocytes B16 (ATCC) using the method of Hosoi et al. with modifications [35]. Cells were seeded at a density of 5000 cells per cm^2^ in DMEM culture medium supplemented with 10% fetal bovine serum and 100 U penicillin-stimulating/100 μg mL^−1^ streptomycin. After 24 h of cultivation, samples were added to the cell cultures and cultivated for 48 h. Cultivation was carried out at 37 °C in a 5% CO_2_ atmosphere. After incubation, cells were detached from the culture surface by treatment with 0.25% trypsin/EDTA for 5 min and rinsed twice with phosphate-buffered saline (pH 7.4), and cell counts were determined with a hemacytometer. The cell suspensions were lysed with 250 μL of previously prepared 1 N sodium hydroxide at 60 °C for 1 h; then, 200 μL of the lysate was transferred to a 96-well microplate, and the optical density was read at 405 nm. Changes in melanin were determined relative to solvent controls. A total of 1 mM kojic acid (Sigma) was used as a positive control. Three replicates were analyzed throughout the study. Average ± standard deviation (SD) was used to express the experimental values. One-way analysis of variance (ANOVA) with Tukey’s multiple comparison test was used for statistical analysis. A *p*-value < 0.05 was considered statistically significant.

## 3. Results

### 3.1. Supercritical Fluid Extraction

Total extraction yield is one of the most important ways to measure the effectiveness of an extraction process. It is especially important to look at the recovery of bioactive compounds when evaluating how to make use of waste streams. The experimental matrix and the total extraction yields are compiled in Table 4.

**Figure 1:**
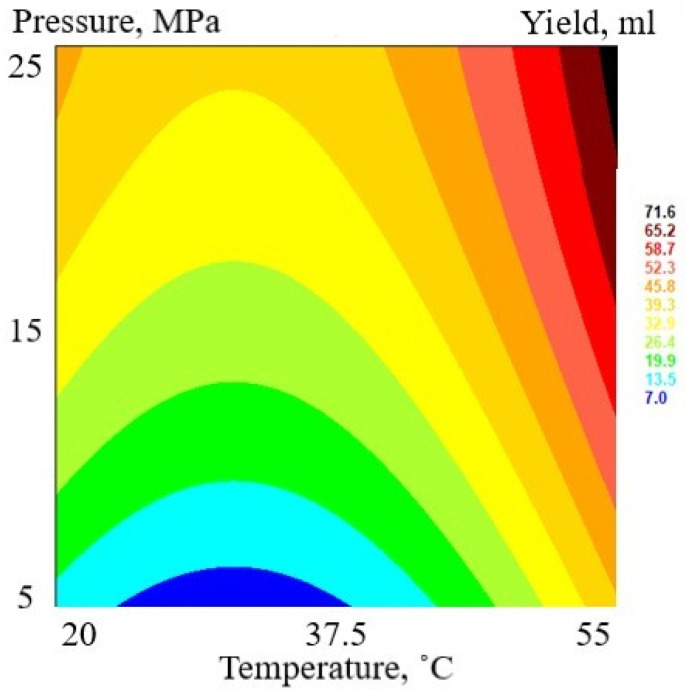
Response surface plot of total extraction yield (mL) as a function of pressure (MPa) and temperature (°C).

The highest total extraction yield (FAF3) obtained with an extraction temperature of 55 °C and pressure of 25 MPa is 14.98 mL 100 g^−1^ DW, and the lowest yield (FAF8) is 1.87 mL 100 g^−1^ DW, obtained under 5 MPa and 20 °C. The response surface methodology was used to evaluate the extraction parameter influence on total extraction yield (Figure 1).

The total extraction yield shows a tendency to increase when both extraction pressure and temperature increase. Both factors are statistically significant (*p* < 0.05).

### 3.2. Phytochemical Screening of Matricaria chamomilla White Ray Floret Supercritical Fluid Extracts

**Figure 2:**
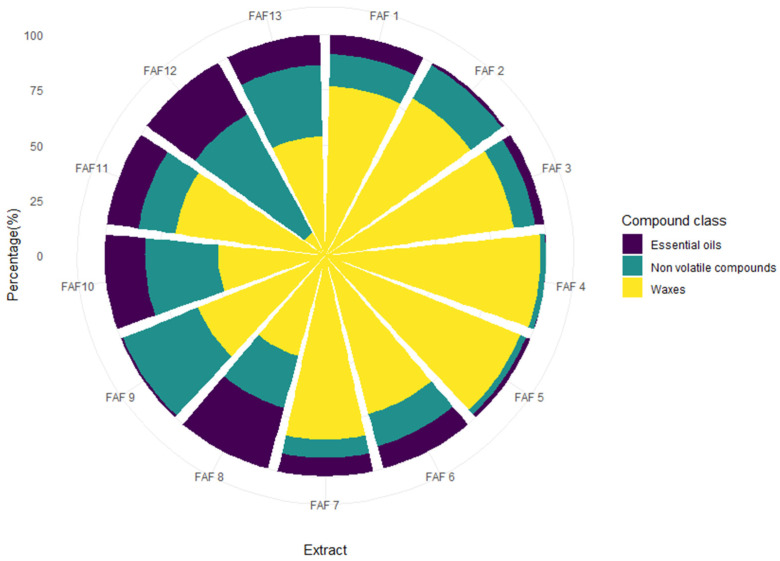
Circular bar plot of mass fraction content of essential oils, waxes, and nonvolatile compounds in *Matricaria chamomilla* white ray floret supercritical fluid extracts.

Screening of phytochemical classes in supercritical fluid (SCF) extracts from chamomile herb processing byproducts was performed using gravimetrical analyses and high-throughput 96-well plate spectrophotometric methods. To visualize the ratio between essential oils, waxes, and nonvolatile compounds within each *Matricaria chamomilla* white ray floret supercritical fluid extract, the obtained amount as *wt%* was plotted in the circular bar plot (Figure 2).

Significant differences in the dominant chemical classes were discovered in all thirteen SCF extracts. Waxes were found to be the most dominant group (yellow plot in Figure 2), ranging from 12.1 to 97.4 percent in the extract. Extracts FAF4 and FAF3 showed the highest amounts of waxes (97.4 and 95.3 percent, respectively), while extract FAF12 contained only around 12.1 percent and was found to be the only extract to have a higher amount of the next largest group, nonvolatile compounds (green plot in Figure 2), ranging from 2.5 to 60.9 percent (FAF4 and FAF12, respectively) within the extract. The smallest contribution in the amount of extract mass fraction comes from essential oils (purple plot in Figure 2), ranging from 0.1 to 28.7 percent in FAF4 and FAF8, respectively.

The estimation of the total phenolic content (TPC) and total tannin content (TTC) in all SCF extracts was carried out through the Folin–Ciocalteu assay, the total flavonoid content (TFC) through the aluminum chlorate assay, and the total content of sugars using the phenol–sulfuric acid colorimetric method, respectively. The quenching activity of 2,2-diphenyl-1-picrylhydrazyl (DPPH) was used to measure the antioxidant power of SCF extracts. The data are presented in Table 5 as the mean standard error of three separate experiments.

Based on the phytochemical screening analysis (Table 5), significant variability was observed in the efficiency of SCFE. Total phenolic compound yield of *Matricaria chamomilla* white ray florets ranged from 9.5 ± 1.5 to 30.6 ± 0.3 mg GAE mL^−1^ in FAF3 and FAF9 extracts, respectively. Similar relationships were observed in the amounts of total flavonoid and tannin content among the extracts tested. It is assumed that if the amounts of TPC, TFC, or TTC are high, then the antiradical activity of these extracts will be significantly higher, respectively. However, significant amounts of total sugars were extracted from the SCF extracts investigated in this study (ranging from 3.7 ± 0.6 to 63.3 ± 1.1 mg GLE mL^−1^), partially affecting the DPPH free radical scavenging activity. The antiradical activity (ARA) in the SCF extracts determined ranged from 0.16 ± 0.04 to 1.04 ± 0.07 mg TE mL^−1^. A more precise effect on antiradical activity is described later in Section 3.3 and Section 3.4 by describing individual volatile and nonvolatile compound amounts. 

**Figure 3:**
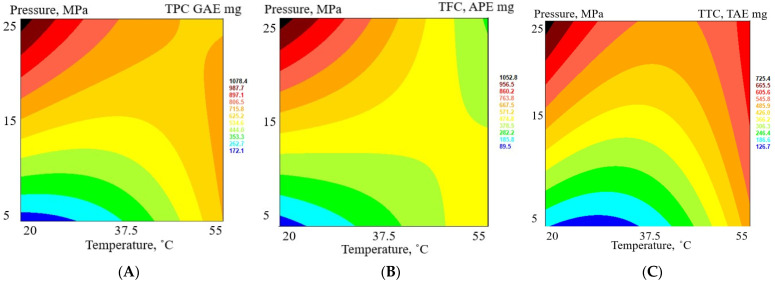

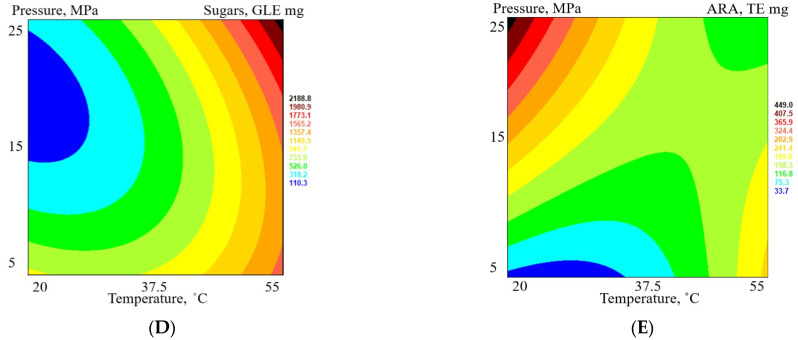
Response surface plots as a function of pressure (MPa) and temperature (°C): (**A**)—total phenolic content, expressed as gallic acid equivalents (GAE mg); (**B**)—total flavonoid content, expressed as apigenin equivalents (APE mg); (**C**)—total tannin content, expressed as tannic acid equivalents (TAE mg); (**D**)—total sugar content, expressed as glucose equivalents (GE mg); and (**E**)—radical scavenging activity, expressed as trolox equivalents (TE mg).

RSM was also used to determine the influence of extraction parameters on the TPC, TFC, TTC, sugars, and ARA of all extracts. Since the extraction yield of each extract is different, the total content of phenolic, flavonoid, tannin, sugar, and antiradical activity of each extract was recalculated in absolute units (mg) so that the total extraction efficiency of these parameters could be compared (see Figure 3 for response surface plots).

The increase in TPC in the extracts is statistically significant with an increase in pressure (an increase from 5 MPa to 25 MPa increases the TPC almost 10-fold). The influence of temperature is not statistically significant (*p* > 0.05). The highest total phenolic content is in extract FAF6 (1209.3 mg GAE), but the lowest is in extract FAF8 (137.9 mg GAE). Similarly, the content of TFC and TTC in extracts increases with the increase in extraction pressure (statistically significant, *p* > 0.05). The impact of extraction temperature on flavonoid and tannin content is not statistically significant. The total flavonoid content in extracts in absolute units varied between 84.7 mg APE (FAF8) and 1154.6 mg APE (FAF6), and the total tannin content in extracts in absolute units was between 107.8 mg TAE (FAF8) and 842.8 mg TAE (FAF6). On the other hand, the increase in total sugar content was statistically significant with an increase in temperature (*p* < 0.05) but not with an increase in pressure (*p* > 0.05). The higher the sugar content in extracts, the lower the content of phenolics, flavonoids, and tannins. The increase in ARA with an increase in extraction pressure was statistically significant (*p* < 0.05), but it was not with temperature (*p* > 0.05).

### 3.3. Quantitative Analysis of the Volatile Components in Matricaria chamomilla White Ray Floret Supercritical Fluid Extracts 

Qualitative analysis of the obtained extracts was performed using GC-MS. The tentative identification of volatile compounds in SCF extracts of *Matricaria chamomilla* white florets was based on the comparison of their mass spectra with spectra from known compounds through a computerized search of the mass spectra in the NIST MS Search 2.2 library. Quantitative analysis was carried out using the external standard (α-bisabolol method). Supplementary Table S1 summarizes the amounts of the 37 identified volatile compounds that were separated (mg mL^−1^ α-bisabolol equivalents). See Figure S1 for GC-MS mass spectra for all identified compounds according to Supplementary Table S1). Generally, the chemical composition of the SCF extracts being studied was quite different, showing an abundance of essential oil components, hydrocarbons, fatty acids, phytosterols, and pentacyclic triterpenes with wide concentration ranges. The main essential oil constituents as detected by GC-MS were cis-ene-yne-dicycloether (0–9.02 mg mL^−1^), α-bisabolol oxide A (0–6.22 mg mL^−1^), α-bisabolol oxide B (0–5.69 mg mL^−1^), and (E)-β-farnesene (0–3.64 mg mL^−1^). Although a clear predominance of these compounds prevailed in extracts FAF12 > FAF10 > FAF13 > FAF6, oxygenated sesquiterpenes and nonterpene compounds were present in all SCF extracts, except FAF4. Fatty acid compounds (V17-V21 in Table S1) were found mainly in extracts FAF10 < FAF13 < FAF12, making up from 1.3 to 3.3 percent of all separated volatile compounds, respectively. When looking at the amounts of hydrocarbons, it is important to note that they are not only found in one extract FAF9. Instead, they are found in the highest amounts in the extract FAF8, which makes up 49.0 percent of all separated volatile compounds. The dominant ones were tetracosane (0–0.54 mg mL^−1^), found in all extracts except FAF8, heptacosane (0–0.96 mg mL^−1^), and hexacosane (0–2.92 mg mL^−1^). The most abundant phytosterol was stigmasterol (V30 in Table S1), which was found in all SCF extracts except FAF4, with levels reaching 3.05 mg mL^−1^ in extract FAF12. It was followed by β-sitosterol (V31) and capesterol (V29), the latter of which could only be isolated from the FAF12 extract. The class of pentacyclic triterpenes is the most dominant among all extracts, ranging from 1.7% (FAF8) to 92.6% (FAF9) of all separated volatile compounds. Lupeol (V32) is the only isolated compound found in the thirteen extracts, compiling one of the highest concentrations in the samples FAF13 < FAF10 < FAF9 < FAF12, followed by lupenone (V35), which is not only found in FAF8 and α-/β-amyrines (V34 and V33). Since lactones and polyphenol compounds have different polarities, they are not often found in essential oils. However, they can be found in extracts made with polar or semipolar solvents, such as ethanol. As evidence, the presence of coumarin herniarin (V12) and lactone matricarin (V22) was confirmed, although only in extract FAF12. However, liquid chromatography analysis methods, which are discussed in the following section, are unquestionably superior for performing this type of analysis.

### 3.4. Quantitative Analysis of the Nonvolatile Components in Matricaria chamomilla White Ray Florets Supercritical Fluid Extracts

The qualitative analysis of the nonvolatile compounds in the obtained SCF extracts was conducted using LC-qTOF-MS analysis in positive ESI+ mode, which allowed the untargeted identification of 61 constituents in total (Supplementary Table S2). Less polar prenol lipids, fatty acids, lignans, and glycerophosphates were found to be the most abundant components of the extracts investigated, followed by more polar flavonoids, amino acids, lactones, etc. It was found that *Matricaria chamomilla* white ray florets are a rich source of apigenin, coumarin, and ferulic acid derivatives in significant quantities. Although the purpose of this study was not to quantify all identified nonvolatile compounds, the amounts of the latter compounds and their presence in the extracts seemed significant for their quantification by external standards. The first important chemical class (compounds AA1–AA6 in Supplementary Table S3) that we isolated was amino acids and their derivatives, whose quantification was performed using leucine. Representatives of this class were found more or less in all extracts being studied, ranging from 0.06 (FAF9) to 10.30 (FAF4) and 11.43 (FAF2) µg mL^−1^ leucine equivalents. The most abundant amino acid was found to be leucine (AA3), followed by phenylalanine (AA5) and homostachydrine (AA2). The next chemical class that was highlighted was ferulic acid derivatives, quantified using ferulic acid (FA1–FA3 in Supplementary Table S3). All three representatives of this group are ferulic acid-O-glucoside isomers, whose presence was found in all extracts except for FAF12. The highest concentrations were found in the extracts of FAF3 > FAF2 > FAF4. Furthermore, coumarin derivatives (COU1-COU4), calculated from coumarin, were found in all extracts, ranging from 6.63 (FAF9) to 50.01 (FAF12) µg mL^−1^ coumarin equivalents. As expected, herniarin (COU3) was the most dominant of all coumarins, followed by umbelliferone (COU2). Both were extracted from all obtained extracts. The last significant chemical class of nonvolatile compounds that was calculated separately was apigenin derivatives (API1-API6), ranging from 0.11 (FAF9) to 48.32 (FAF2) µg mL^−1^ apigenin equivalents. The most important representatives of this class should be noted: apigenin-7-O-glycoside (API1), which found its place in the European Pharmacopoeia, as well as apigenin (API6) and its glucosides. See LC-HRMS mass spectra for identified compounds according to Supplementary Table S3 in Supplementary Figure S3.

### 3.5. Antimicrobial Activity

Six samples were selected for further analysis of biological activity. The selection was based on the total phenolic and flavonoid content, as well as antiradical activity and total content of coumarins, ferulic acid derivatives, apigenin derivatives, and volatile compounds. To elucidate the potential roles of different compounds on biological activity, extracts were selected to be different in their chemical composition. Selected samples were tested for their antimicrobial activity against bacteria *S. aureus*, *S. epidermidis*, *E. coli*, *P. aeruginosa*, and yeast *C. albicans* (Table 6).

Among the extracts tested, FAF2 showed the highest antimicrobial activity. It inhibited the growth of all selected microorganisms and was the most active against *S. aureus* and *S. epidermidis*. Slightly higher MIC values against Gram-positive bacteria were detected in samples FAF7, FAF10, and FAF12. The MBC values of FAF7, FAF10, and FAF12 were the lowest against *S. aureus*. FAF7 and FAF12 were the least effective against Gram-negative bacteria. FAF7 did not show any effect against *E. coli* and was not bactericidal against *P. aeruginosa*, whereas FAF12 showed antimicrobial activity only at high concentrations. All the extracts were fungistatic against *C. albicans*. 

FAF2 differs from other extracts with its high content of apigenin and its derivatives. FAF10 and FAF12 contain high concentrations of coumarin derivatives. All three were rich in lupenone. These differences might be responsible for variations in antimicrobial activity [36,37,38,39]. It is of special interest that all the samples inhibit the growth of *C. albicans*. The ability to simultaneously inhibit staphylococcal and yeast growth makes these extracts interesting for skin care applications, where the regulation of the skin microbiome, including inhibition of the growth of pathogens and opportunistic pathogens, is important.

### 3.6. Cytotoxicity and Phototoxicity

**Figure 4:**
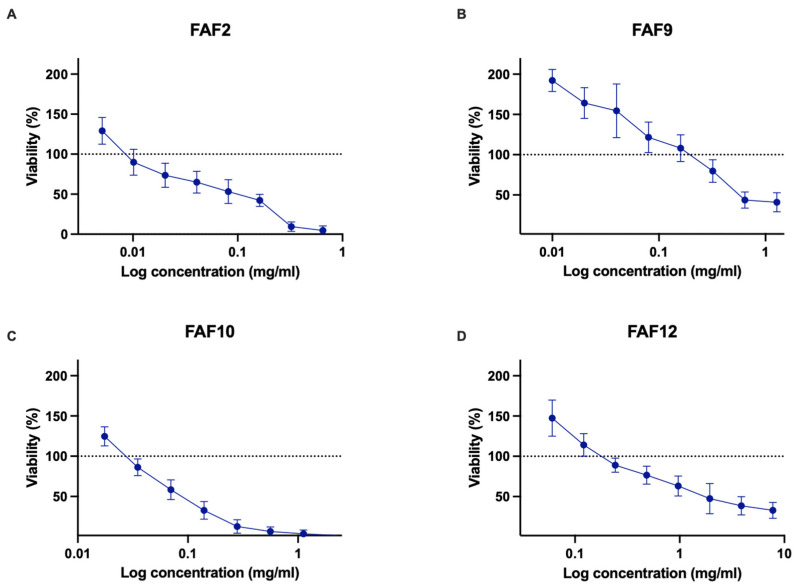
Cytotoxicity of samples in Balb/c 3T3 cell line with results expressed as a relative change compared with untreated control: (**A**)—FAF2; (**B**)—FAF9; (**C**)—FAF10; and (**D**)—FAF12. The dotted line represents the untreated control level; data shown as mean ± SD (*n* = 3).

Samples FAF2, FAF9, FAF10, and FAF12 were chosen for further analysis based on their antimicrobial activity and chemical composition data. Cytotoxicity testing data are shown in Figure 4.

All tested samples showed decreasing cell viability with increasing extract concentrations. In the concentration range of 0.005–0.02 mg mL^−1^, FAF2 did not show significant cytotoxic effect—cell viability did not decrease by more than 27% (Figure 4A). However, concentrations greater than 0.325 mg mL^−1^ decreased viability by more than 90%. Interestingly, the lowest concentration tested, 0.005 mg mL^−1^, promoted cell proliferation on an average of 29.18 ± 16.62%. FAF9 did not reduce cell viability in the concentration range of 0.01–0.32 mg mL^−1^ but was cytotoxic at concentrations greater than 0.64 mg mL^−1^ (Figure 4B). This extract had a concentration-dependent cell proliferation stimulating effect on Balb/c 3T3 cells. A statistically significant increase in cell proliferation (*p* < 0.05) was observed in the concentration range of 0.01–0.04 mg mL^−1^. In the presence of 0.01 mg mL^−1^, proliferation almost doubled (192.15 ± 13.67% compared with control). FAF10 was not cytotoxic in the concentration range of 0.018–0.035 mg mL^−1^ but showed negative effects on cell viability at higher concentrations (Figure 4C). The lowest concentration tested (0.018 mg mL^−1^) had slightly stimulated activity—cell proliferation increased by 24.70 ± 11.80%. FAF12 was cytotoxic at concentrations greater than 0.97 mg mL^−1^ (Figure 4D); the lowest tested concentration had a positive effect on cell viability and proliferation—viable cell count increased by 47.50 ± 22.46%.

When looking at cytotoxicity data in conjunction with antimicrobial activity data, it is important to note that the concentrations of FAF9 and FAF12 that inhibit Gram-negative bacteria and *C. albicans* from growing fall in the range of concentrations that do not cause cytotoxicity. Extract FAF2 and FAF10 concentrations that show antimicrobial activity are cytotoxic in mammalian cell culture.

**Figure 5:**
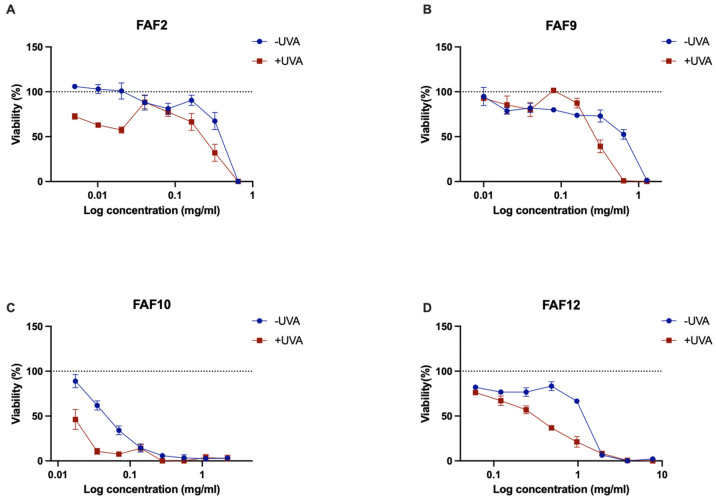
Phototoxicity of samples in HaCaT cell line with results expressed as a relative change compared with untreated control: (**A**)—FAF2; (**B**)—FAF9; (**C**)—FAF10; and (**D**)—FAF12. The dotted line represents the untreated control level; data shown as mean ± SD (*n* = 3).

Phototoxicity was tested in the human keratinocyte cell line HaCaT (Figure 5). Data indicate the potential phototoxicity of samples FAF2, FAF10, and FAF12. Sample FAF2 reduced keratinocyte viability in the presence of UVA irradiation, even at the lowest tested concentrations, compared with nontreated and nonirradiated cells (Figure 5A). FAF9 did not change cell viability, irradiated or not, in a concentration range of 0.01–0.16 mg mL^−1^, while higher concentrations after UVA irradiation significantly reduced viable cell counts compared with samples that were not irradiated (Figure 5B). Even at the lowest concentration tested, FAF10 reduced keratinocyte viability by more than 53% if treated with UVA (Figure 5C). The reduction in cell viability in the presence of FAF12 was more pronounced if the cells were treated with UVA (Figure 5D).

The cytotoxic effects of FAF2, FAF10, and FAF12 could be explained by the high content of coumarin derivatives.

### 3.7. Effect on Melanocytes

**Figure 6:**
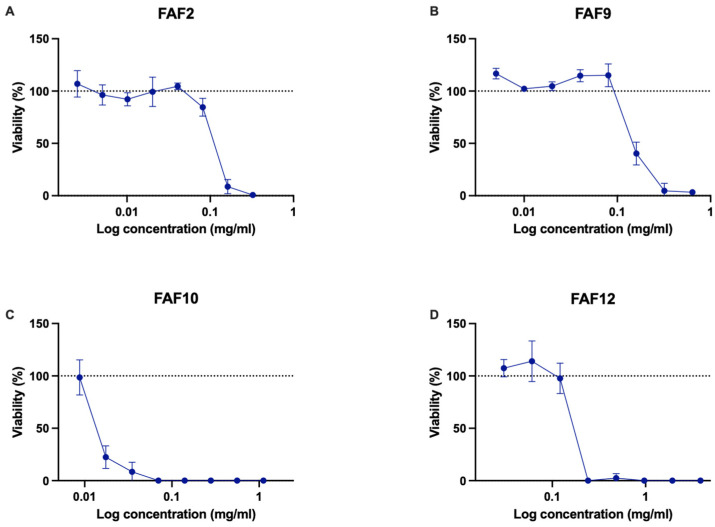
Cytotoxicity of samples in B16 cell line with results expressed as a relative change compared with untreated control: (**A**)—FAF2; (**B**)—FAF9; (**C**)—FAF10; and (**D**)—FAF12. The dotted line represents the untreated control level, data shown as mean ± SD (*n* = 3).

As some of the dominant compounds in the samples have skin-pigmentation-regulating activities, selected samples were tested in melanocyte cell cultures. Initially, the murine melanocyte cell line B16 was used to assess the effects on the cell viability. Samples FAF2 and FAF9 did not change cell viability and proliferation at lower concentrations (Figure 6A,B). Cell viability was greater than 80% in the concentration range of 0.003–0.081 mg mL^−1^ for FAF2, and a slight cell-proliferation-stimulating effect was observed in the concentration range of 0.005–0.08 mg mL^−1^ for FAF9. In the presence of FAF10, cell viability decreased significantly at concentrations greater than 0.018 mg mL^−1^ (Figure 6C). At concentrations of 0.121 mg mL^−1^ and below, the viability of melanocytes was not affected by FAF12, while higher concentrations reduced cell viability by more than 95% (Figure 6D).

The observed effect might be explained by the high content of coumarins in samples FAF10 and FAF12, as well as the content of volatile compounds.

**Figure 7:**
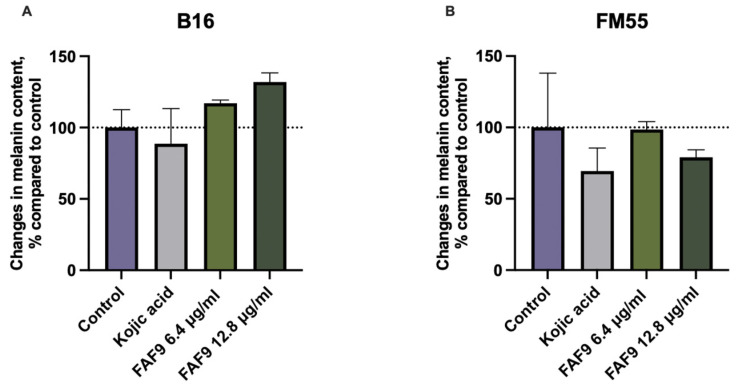
Changes in melanin content in B16 (**A**) and FM55 (**B**) melanocytes. Results expressed as a relative change compared with untreated control. The dotted line represents the untreated control level; data shown as mean ± SD (*n* = 3).

Among the selected samples, FAF9 had the highest content of TFC and TPC, and at low concentrations, it did not have a negative effect on cell viability. It also showed a good safety profile in cytotoxicity and phototoxicity assays, and it was tested for the ability to reduce melanin production. The effect on melanin production was assayed in two cell lines: murine melanocytes (B16) and human melanocytes (FM55). The effects differed between cell lines, with FM55 melanocytes being more responsive to the positive control, kojic acid, as well as FAF9 (Figure 7). In FM55 incubation, the highest tested concentration (0.0128 mg mL^−1^) reduced the melanin content by 24.53%. Lower concentrations did not have an effect on melanin production in FM55 cells. In contrast, both concentrations tested increased melanin content in the B16 cell line. This result points to the different responsiveness of the melanocyte cell lines. The effect of the positive control, kojic acid, also differed among cell lines.

## 4. Discussion

For the application of any new technology or the use of new materials, the quantification and characterization of the results are a critical point to being able to evaluate how the technology is performing compared with traditional techniques and methods. The extraction of bioactive compounds from medicinal and aromatic plants is a research area essential for their application to the pharmaceutical, food, or cosmetic industries. 

In this study, supercritical fluid extraction of the *Matricaria chamomilla* processing waste stream of white ray florets was carried out. The highest obtained extract yield was 14.98 mL 100 g^−1^ DW at 25 MPa pressure and 55 °C temperature, with CO_2_ flow of 100 L h^−1^, ethanol as cosolvent flow of 4 mL min^−1^, and an extraction duration of 2 h. The highest overall extraction yield is not as high as previously reported by Nakurte [30] using *M. chamomilla* white ray florets and fractional SFE. Other reported values for German chamomile flowerhead SFE are between 0.23–3.64 g 100 g^−1^ by Molnar and his team [40], 2.5–3.8 g 100 g ^−1^ by Kotnik [41], and 9.2–9.7 g 100 g^−1^ by Scalia [42]. Povh et al. [43] have reported extraction yields of up to 4.3 g 100 g^−1^. Only three extracts (FAF3, FAF5, and FAF6) have higher yields than previously reported. 

Previous research by Scalia [42] observed that there was no improvement in extraction efficiency by changing the extraction temperature (40–50 °C, at 20 MPa), but Kotnik [41] has reported an increased extraction yield with increased extraction temperature (30–40 °C, at 10–25 MPa). Our results are in line with those of Povh [43] and show that increases in both extraction pressure and temperature increase the total extract yield. Povh [43] and Al-Soud [44] highlight that other extraction parameters, such as CO_2_ flow rate and cosolvent concentration, also impact the total extraction yield.

**Figure 8:**
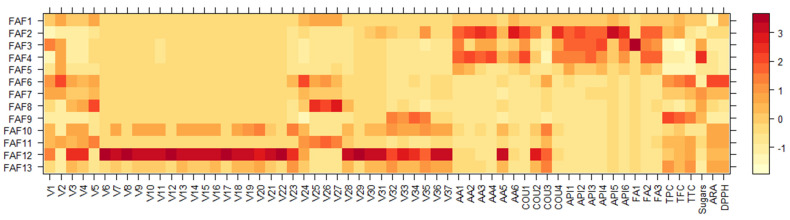
Variation in chemical composition of *Matricaria chamomilla* white ray floret supercritical fluid extracts: the legend denotes scaled values of the volatile chemical constituents (V1-V37, according to Table S1), general nonvolatile constituents (AA1–AA6; COU1-COU4; and API1-API6, according to Table S3), and total content of phenolics (TPC), flavonoids (TFC), tannins (TTC), sugars, and antiradical (ARA) (according to Table 5).

The use of SCFE results in a high yield of various bioactive compounds from *Matricaria chamomilla* white ray florets. Quantitative amounts of separated compounds are not comparable with those found in other studies because they depend on several factors—the part of the chamomile plant used, the country of origin, the variety, the processing of the material, and the extraction methods. However, by summarizing the studies conducted in the past, the groups of dominant compounds are mostly the same. These naturally occurring organic compounds can act as antioxidants by transferring electrons, and they could be mostly responsible for the antioxidant and biological activities of the extracts that were tested. Figure 8 shows the results of a statistical analysis using a heatmap dendrogram. The obtained dendrogram clearly indicates the differences between the extracts obtained in terms of the amounts of individual chemical compounds present in them. Extracts FAF1–FAF5 contained more groups of nonvolatile compounds, while FAF6–FAF13 were clearly dominated by groups of volatile compounds. In addition to nonvolatile compounds, in extracts FAF10, FAF12, and FAF13, a high amount of coumarin derivatives was detected.

Since the supercritical fluid extraction of chamomile has been reported in a number of studies [42,43,45,46], it was already known that CO_2_ extracts can contain essential oil components. Their lipophilic nature and low molecular weight make it easier for them to get through the cell membranes. After their entry into cells, they can exhibit various biological effects, including antimicrobial [14,47], antiviral [3,48,49], and anti-inflammatory activity [7,48,50].

Among all separated components, analysis of volatile compounds in *Matricaria chamomilla* white ray floret SCF extracts revealed essential oil representatives, such as terpenoids, spiroethers, and tetrahydrofurans, which constituted 2.67–38.56% of the extracts. With the exception of FAF4, all SCF extracts contained tehrahydrofuranes, α-bisabolol oxides A and B, and α-bisabolone oxide A (V9, V11, and V13 in Figure 8, respectively), which have important pharmacological and cosmetic applications [50,51] and are listed in the European Pharmacopoeia. α-bisabolol and its derivatives alleviate skin inflammation, and they have good skin absorption properties and safety profiles; therefore, they are popular ingredients that are included in various skincare products [52,53]. It was observed in previous findings [41] that the highest concentration of α-bisabolol oxides in SFE extracts was under 25 MPa and 30 °C and was not directly correlated with extraction temperature and pressure. This falls in line with our findings, where the optimal extraction parameters for α-bisabolol oxide extraction are 15 MPa and 37.5 °C. Due to their biological activities, it is also important to mention the presence of polyacetylenic compounds, called spiroethers, such as cis-ene-yne-dicycloether (V14), E-en-yn-dicycloether (V15), and (E)-tibetin spiroether (V16), as well as the presence of (E)-β-farnesene (V3) [48,50,54].

Our results clearly show that different operating conditions for supercritical fluid extraction have a great effect on the chemical profile of the extracts, as was seen in earlier studies [30,41,42,55,56]. For example, higher operating pressures can produce more high-molecular-weight chemicals, such as waxes [57,58]. Several of the identified aliphatic hydrocarbons (V23-V27) and phytosterols (V29-V31) are described as possessing antibacterial and antifungal activities [43,49,50]. Zengin [59], in very recent research, confirmed that hydrocarbons such as tetracosane (V24) and hexacosane (V25) extracted by SCFE from chamomile are strongly correlated with the inhibitory effect of cholinesterase. In contrast to paraffins derived from fossil components, odd-carbon-atom hydrocarbons are significantly more biocompatible with human skin and can be used in cosmetics and pharmaceuticals [58,60]. Although several SCF extracts showed an extremely high level of triterpenes (V32-V37), which are not often described as being found in the *Matricaria-chamomilla*-derived extracts, α- and β-amyrine (V34 and V33), which belong to the pentacyclic triterpene class, are notable for the wide range of therapeutic effects they can produce, including anti-inflammatory, antimicrobial, antifungal, and antiallergic properties [61,62]. Triterpenes reduce skin inflammation, alleviate the negative effects of UV exposure, and promote skin regeneration [63].

The use of a cosolvent in a supercritical fluid extraction, such as ethanol, can enhance the extraction yield of polar compounds by increasing the solubility of antioxidants such as phenolic compounds, flavonoids, phenolic diterpenes, etc. [40,64,65]. Apigenin (API6 in Figure 8) is the main bioactive component and is therefore considered a chamomile quality indicator. Apigenin builds up as bound forms of apigenin 7-O-glucoside (API1) and other acylated derivatives in the white ligulate florets of the chamomile anthodium, while mono- and diacetyl glucosides of apigenin undergo rapid ester hydrolysis, resulting in the formation of apigenin 7-O-glucoside [66]. According to the European Pharmacopoeia, *Matricaria chamomilla* flowers must contain at least 0.25 percent apigenin-7-O-glucoside to be used as a therapeutic agent. On the basis of previous studies, it is known that apigenin and its derivatives influence various cellular processes, including cell cycle progression. By stopping cell growth and the expression of oncogenes, chamomile may help with chemoprevention, neuronal activity, and reducing inflammation [67,68]. When applied topically, apigenin downregulates inflammatory mediators and reduces inflammation in atopic skin [69,70,71], and it can alleviate inflammation in psoriatic and eczematic skin. In vitro tests have shown that apigenin can reduce allergic reactions and improve skin barrier function [72]. *Matricaria chamomilla* white floret SCF extracts showed that they contained a mixture of one glucoside (API1), two monoacetyl glucosides (API2 and API3), and two monoacetyl/monomalonyl glucosides (API4 and API5). These findings support previous research by Švehlíková [66], who discovered different forms of apigenin glucosides in the white florets of *Matricaria chamomilla*, and Tsivelika [73], who discovered different chamomile varieties in Greece. It was observed in previous findings that an increase in extraction pressure increases apigenin and its glucoside extraction [55]. Our findings are in line with this, showing a statistically significant impact of extraction pressure increases the extraction yield of apigenin and its glucosides. As apigenin is reported to be poorly soluble in pure carbon dioxide at 40 °C and 20 MPa [55], the addition of cosolvents such as ethanol is a critical point in its extraction.

Plant coumarins, often called phytoalexins, are believed to be compounds that help plants defend themselves when living and nonliving things cause stress [74]. The literature previously described the enzymatic breakdown of umbelliferone (COU2) to its glucosylated (skimmin (COU1)) and methylated (herniarin (COU3)) derivatives [75]. Our results support what other studies have found: higher concentrations of herniarin than umbelliferone can be extracted from chamomile flower heads [40,76]. Herniarin was found in all thirteen SCF extracts tested in our study, ranging from 1.62 to 43.28 µg mL^−1^ of coumarin *equivalents*, while umbelliferone exceeded concentrations from 0.28 to 6.53 µg mL^−1^, respectively. The higher coumarin and herniarin content can be extracted with higher pressure, which is in line with current research in chamomile [40] and other medicinal plants [77]. Coumarin derivatives are described to have antimicrobial and anti-inflammatory activity [50,78], while umbelliferone absorbs ultraviolet light at multiple wavelengths [79]. Therefore, they are of interest for their application in pharmaceutical and cosmetic products [40]. In in vivo models of atopic dermatitis, coumarins reduced inflammation and promoted skin regeneration [80]. Some coumarin derivatives have been identified as tyrosinase inhibitors, pointing to their potential applications in the regulation of skin pigmentation [81]. Simultaneously, several studies also reveal the potentially negative effects of coumarin and its derivatives on skin. These compounds have irritating and phototoxic activities [82,83]. This is in line with our findings: extracts with higher coumarin content (FAF10, FAF12) showed greater cytotoxicity and phototoxicity. Umbelliferone is an antioxidant that has anti-inflammatory, UV-protecting, and antimicrobial activities [84,85]—properties interesting not only for medical but also cosmetic applications.

Although there is written information about how to obtain pure ferulic acid from different chamomile products [86,87], we were able to separate only ferulic-acid-related O-glycoside derivatives (FA1, FA2, and FA3). The presence of ferulic-acid-related glucosides in chamomile methanolic extracts was first mentioned by Mulinacci et al. [88], followed by Lin [89] and Tsivelika [73] in German chamomile samples. These glycosides were found in all SCF extracts except FAF12, while extracts FAF2 and FAF3 contained the highest amount of them, up to 14.74 µg mL^−1^ of ferulic acid equivalents, respectively. According to Sun [90], both the increase in extraction pressure and temperature enhanced ferulic acid and its glucoside extraction, which is in an agreement with our findings. As a representative of hydroxycinnamates, ferulic acid derivatives serve as a defense microorganism, as well as a response to UV-B radiation and mechanical damage [91]. According to Turghun [92], certain forms of ferulic acid glycosides showed antioxidant activity, a potent inhibitory effect on protein tyrosine phosphatase (1B PTP1B), and antibacterial activity, with moderate inhibitory effects against *C. albicans*. Ferulic acid is widely used in cosmetics because of its strong antioxidant properties. It efficiently alleviates the negative effects of UV-induced oxidative stress [93,94]. In vivo studies have confirmed ferulic acid skin firming, moisturizing, and pigmentation regulating activity [95].

Primary metabolites, such as amino acids and sugars, are used to check the quality of herbal products and are also useful as secondary plant metabolites. Based on previous findings, these groups are not rare in *Matricaria chamomilla* samples [25,44,50,91]. Al-Soud has noted that extraction temperature increase has a more significant impact on sugar extraction than extraction pressure increase [44]; this is also confirmed by our findings. Nakurte et al. [30], in previous studies, found that the amount of total sugars was highly positively correlated with inhibitory activity against *E. coli* and *P. aeruginosa*, with a weaker influence on inhibitory activity against *S. aureus*.

In general, the tested extracts (FAF2, FAF6, FAF7, FAF9, FAF10, and FAF12) were more active against Gram-positive bacteria than against Gram-negative bacteria. German chamomile extracts have been previously reported to possess antimicrobial activity against *S. aureus*, *E. coli*, and *C. albicans* [13]. The lower activity of plant-derived phenolic substances against Gram-negative bacteria can be attributed to differences in their cell walls, specifically the outer membrane, which blocks the penetration of antimicrobial compounds. However, studies have shown that susceptibility to phenolic compounds varies between bacterial species and even strains and depends on the specific characteristics of the compounds. While it is proposed that the activity against Gram-negative and Gram-positive bacteria may differ due to membrane disruption, there is no clear correlation between Gram staining and susceptibility to secondary plant metabolites [96].

**Figure 9:**
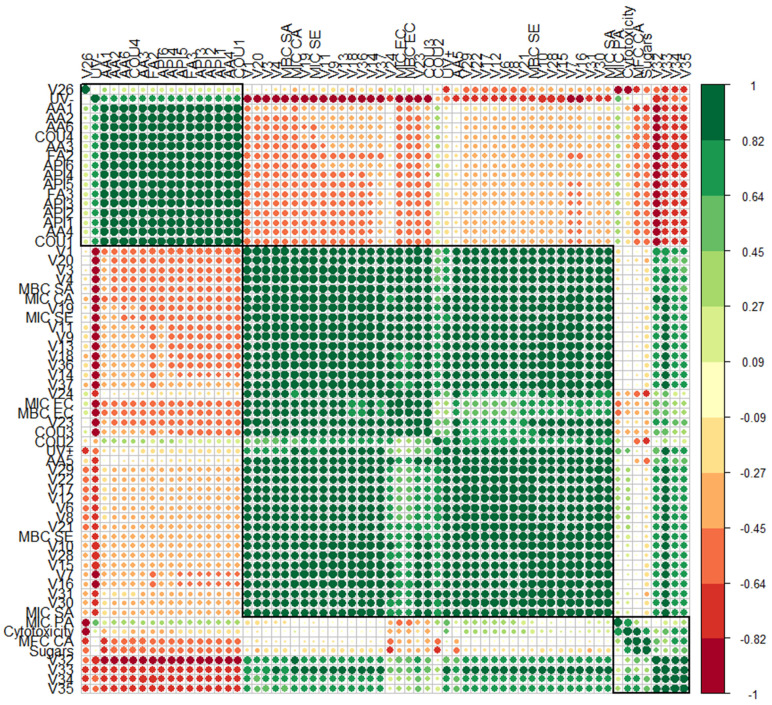
Correlogram depicting the relationship between both volatile compound (V1-V37, in Table S1) and nonvolatile (Table S3) compound concentration and antimicrobial activity (Table 6), as well as cytotoxicity and phototoxicity (Figure 4, Figure 5 and Figure 6): correlations with *p* > 0.05 are considered insignificant and are denoted by a white space with a blank. The color and size of the squares are proportional to the correlation coefficients, which are color-coded from deep red (−1) to deep green (1).

To confirm the degree of correlation between the specified variables, a correlogram was created (Figure 9).

The analysis presented in Figure 9 demonstrates that there is a positive correlation between the phototoxicity parameters (UV− and UV+) and the extracted phytochemicals. Nonvolatile compounds, such as amino acids (excluding AA5), apigenin, coumarin (excluding COU2 and COU3), and ferulic acid derivatives, showed a positive correlation with UV−. Meanwhile, almost all volatile compounds (except terpenes V32–V35), two coumarin derivatives (COU2 and COU3), and an amino acid (phenylalanine or AA5), displayed a positive correlation not only with UV+ but also with minimal bactericidal concentration (MBC) against *S. aureus*, *S. epidermidis*, and *E. coli*, as well as minimal inhibitory concentrations (MIC) against *S. aureus, S. epidermidis, E. coli*, and yeast *C. albicans*, indicating that our extracts have potential as antimicrobial agents. Four terpenes (V32–V35) and all sugars, located in the smallest cluster, were positively correlated with minimal fungicidal concentration (MFC) against yeast *C. albicans*, MIC against *P. aeruginosa*, and cytotoxicity, which means they have the ability to induce cell death or damage. These findings confirm that the compounds derived from our extracts can help repair damaged skin cells, improve skin barrier function, and provide further protection to skin from phototoxic damage.

## 5. Conclusions

The waste stream of white ray florets from *Matricaria chamomilla* was extracted using supercritical fluid extraction (SCF) with ethanol as a cosolvent under different conditions. The effects of extraction temperature and pressure on the extraction yield and extract content of total phenolic content (TPC), total flavonoid content (TFC), total terpenoid content (TTC), sugars, and antiradical activity (ARA) were evaluated using response surface methodology (RSM). The results indicate that both extraction temperature and pressure influenced the extraction yield, while only extraction pressure influenced the TPC, TFC, TTC, and ARA of extracts, and only extraction temperature influenced the extracted sugar content. The extracts obtained were chemically characterized, and their antioxidant activity was determined. The results reveal that the extraction conditions altered the chemical profiles and biological abilities of the extracts.

Furthermore, antimicrobial activity was determined for 6 of the 13 selected extracts against *S. aureus*, *S. epidermidis*, *E. coli*, *P. aeruginosa*, and *C. albicans*. Cytotoxicity (in the Balb/c 3T3 cell line) and phototoxicity (in the human keratinocyte cell line HaCaT) were determined for four of six selected extracts. Two of these extracts demonstrated that the concentrations required to inhibit Gram-negative bacteria and *C. albicans* fell within the noncytotoxic concentration range. Both samples were extracted with 15 MPa pressure at 37.5 °C and showed the greatest promise for application in cosmetic products. In conclusion, our findings confirm that the compounds extracted from the *Matricaria chamomilla* waste stream of white ray florets can be used to repair damaged skin cells, improve skin barrier function, and offer further protection to the skin from phototoxic damage.

## Figures and Tables

**Table 1:** Conditions and levels of two independent variables in factorial design of SFE experiments.

Variable	Factor Levels		
	−1	0	1
Pressure (MPa)	5	15	25
Temperature (°C)	20	37.5	55

**Table 2:** Summary of the procedures used in the phytochemical screening of secondary metabolites.

Carried Test	Procedure	Wavelength	Standard Concentration Range
Total phenolic content (TPC)	A total of 25 µL of extract was mixed with 75 µL of H_2_O and 25 µL of Folin–Ciocalteu reagent (1:10) for 6 min. Then, 100 µL of a 7% Na_2_CO_3_ solution was added; the plate was shaken for 30 s and left in a dark place at room temperature for 90 min.	765 nm	0.025–0.20 mg mL^−1^ gallic acid solutions
Total flavonoid content (TFC)	A total of 20 µL of extract was mixed with 15 µL of 5% NaNO_2_ solution for 5 min. Then, 15 µL of a 10% AlCl_3_ solution was added, and the reaction time was 6 min. Then, 100 µL of a 1 M NaOH solution was added. The plate was shaken for 30 s before being placed in a dark, room-temperature environment for 15 min.	510 nm	0.025–0.2 mg mL^−1^ apigenin solutions
Total tannin content (TTC)	A total of 50 µL of extract was mixed with 50 µL H_2_O and 50 µL Folin–Ciocalteu reagent (1:1 dilution); reaction time was 5 min. Then, 100 µL of a 35% Na_2_CO_3_ solution was added, the plate was shaken for 30 s, and it was left in a dark place at room temperature for 30 min.	700 nm	0.01–0.15 mg mL^−1^ tannic acid solutions
Total sugar content (Sugars)	A total of 50 µL of extract was mixed with 150 µL of H_2_SO_4_ and 30 µL of 5% phenol reagent before being heated in an oven at 90 °C for 5 min. After heating, the plates were cooled.	490 nm	0.045–0.90 mg mL^−1^ glucose solutions
Antiradical activity/DPPH radical scavenging (ARA/DPPH)	A total of 20 µL of extract was mixed with 180 µL of 150 µM DPPH reagent. The plate was kept in the dark at room temperature for 60 min.	517 nm	0.018–0.22 µg mL^−1^ trolox solutions

**Table 3:** Prepared targeted individual standards for individual and compound quantification.

Compound	Class	Purity	Mass, mg	Volume, mL	Stock Solution, mg mL^−1^	Calibration Range
Leucine	Amino acids	99%	9.8	10 mL water	0.98	1–50 μg mL^−1^ R^2^ = 0.996
Ferulic acid	Phenolic acids	98%	10.1	10 mL methanol	1.01	1–100 μg mL^−1^ R^2^ = 0.9999
Apigenin	Flavonoids	99.08%	9.0	100 mL ethanol	0.09	100–100,000 ng mL^−1^ R^2^ = 0.9997
Coumarin	Hydroxycoumarins	98%	9.8	10 mL methanol	0.98	100–100,000 ng mL^−1^ R^2^ = 0.997

**Table 4:** Experimental matrix and SCFE yield expressed in mL 100 g^−1^ DW.

Run	Sample	Temperature (°C)		Pressure (MPa)		Yield
		C	U	C	U	mL 100 g^−1^ DW
1	FAF1	0	37.5	0	15	5.62
8	FAF2	0	37.5	1	25	5.62
6	FAF3	1	55	1	25	14.98
13	FAF4	0	37.5	−1	5	3.75
7	FAF5	1	55	0	15	11.24
3	FAF6	−1	20	1	25	9.36
12	FAF7	1	55	−1	5	7.49
4	FAF8	−1	20	−1	5	1.87
2	FAF9	0	37.5	0	15	4.68
10	FAF10	0	37.5	0	15	3.75
11	FAF11	−1	20	0	15	5.62
5	FAF12	0	37.5	0	15	7.49
9	FAF13	0	37.5	0	15	5.62

C—coded; U—uncoded.

**Table 5:** Total content of phenolics, flavonoids, tannins, sugars, and DPPH free radical scavenging activity of *Matricaria chamomilla* white ray floret supercritical fluid extracts.

Extract	TPC ^a^, GAE mg mL^−1^	TFC ^b^, APE mg mL^−1^	TTC ^c^, TAE mg mL^−1^	Sugars ^d^, GLE mg mL^−1^	ARA ^e^, TE mg mL^−1^	DPPH ^f^ Quenched, %
FAF1	18.7 ± 1.8	16.2 ± 1.1	12.6 ± 1.8	21.7 ± 1.1	0.57 ± 0.07	31.6 ± 1.3
FAF2	19.3 ± 1.2	19.1 ± 0.5	10.2 ± 1.7	5.8 ± 0.4	0.38 ± 0.08	21.6 ± 0.9
FAF3	9.5 ± 1.5	4.3 ± 0.6	9.3 ± 1.7	36.9 ± 2.2	0.17 ± 0.05	10.6 ± 0.5
FAF4	11.5 ± 1.5	8.7 ± 1.9	8.1 ± 1.0	63.3 ± 1.1	0.21 ± 0.06	13.1 ± 0.4
FAF5	9.7 ± 1.1	5.1 ± 0.4	7.7 ± 1.5	12.7 ± 1.2	0.24 ± 0.02	14.5 ± 0.6
FAF6	24.2 ± 2.0	23.1 ± 0.2	16.9 ± 1.1	3.7 ± 0.6	1.04 ± 0.07	55.5 ± 1.4
FAF7	17.6 ± 2.4	14.1 ± 2.1	12.7 ± 2.1	42.7 ± 1.6	0.57 ± 0.09	31.5 ± 0.8
FAF8	13.8 ± 1.7	8.5 ± 1.8	10.8 ± 2.3	34.8 ± 1.9	0.31 ± 0.05	18.0 ± 0.3
FAF9	30.6 ± 0.3	25.3 ± 2.6	16.5 ± 3.0	29.0 ± 1.6	0.16 ± 0.04	10.2 ± 0.2
FAF10	15.6 ± 0.2	15.3 ± 0.9	10.1 ± 2.0	11.6 ± 2.0	0.66 ± 0.05	36.2 ± 0.7
FAF11	19.0 ± 1.9	15.8 ± 1.0	13.8 ± 1.7	19.2 ± 1.2	0.67 ± 0.06	36.7 ± 0.6
FAF12	18.0 ± 2.1	16.9 ± 2.3	13.9 ± 2.1	12.3 ± 2.0	0.57 ± 0.08	31.6 ± 0.4
FAF13	23.3 ± 2.5	15.9 ± 1.2	14.9 ± 2.2	7.0 ± 0.7	0.61 ± 0.06	33.4 ± 1.2

^a^ Total phenolic content is expressed as the gallic acid equivalents per milliliter of extract (mg GAE mL^−1^). ^b^ Total flavonoid content is expressed as the apigenin equivalents per milliliter of extract (mg APE mL^−1^). ^c^ Total tannin content is expressed as the tannic acid equivalents per milliliter of extract (mg TAE mL^−1^). ^d^ Total sugar content is expressed as the glucose equivalents per milliliter of extract (mg GLE mL^−1^). ^e^ ARA radical scavenging activity is expressed as the trolox equivalents per milliliter of extract (mg TE mL^−1^). ^f^ DPPH radical scavenging activity of 1% extracts (on a dry basis) is expressed in %.

**Table 6:** Minimal inhibitory concentrations (MIC) and minimal bactericidal (MBC) or minimal fungicidal (MFC) concentrations of selected extracts, mg mL^−1^.

Extract	*S. aureus*		*S. epidermidis*		*E. coli*		*P. aeruginosa*		*C. albicans*	
	MIC	MBC	MIC	MBC	MIC	MBC	MIC	MBC	MIC	MFC
FAF2	0.051	0.203	0.051	0.051	0.813	0.813	0.203	0.406	0.203	0.813
FAF6	0.097	0.388	0.388	0.388	0.775	0.775	0.194	n	0.194	0.194
FAF7	0.045	0.181	0.181	0.181	n	n	0.091	n	0.091	0.362
FAF9	0.20	0.40	0.10	0.10	1.60	1.60	0.40	0.80	0.20	0.20
FAF10	0.088	0.088	0.044	0.088	1.40	1.40	1.40	1.40	0.175	1.40
FAF12	0.076	0.151	0.076	0.078	4.844	4.844	2.422	4.844	0.303	2.422
Solvent control (70% ETOH)	25	n	6.25	25	12.5	12.5	12.5	25	12.5	n

n—antimicrobial activity was not detected.

## Data Availability

The data presented in this study are available on request from the corresponding author.

## Supplementary Materials

The following supporting information can be downloaded at: https://www.mdpi.com/article/10.3390/antiox12051092/s1, Table S1: Chemical composition of separated volatile compounds from *Matricaria chamomilla* white ray floret supercritical fluid extracts; Figure S1: GC-MS mass spectra for all identified compounds in *Matricaria chamomilla* white ray floret supercritical fluid extracts according to Supplementary Table S1. Table S2: Tentative phytocomponents, extracted from the *Matricaria chamomilla* white ray floret supercritical fluid aqueous ethanol extracts, detected by LC-qTOF-MS; Table S3: LC-qTOF-MS data of selected nonvolatile compounds detected in *Matricaria chamomilla* white ray floret supercritical fluid extracts in positive ionization mode. Figure S2: LC-HRMS mass spectra for identified compounds in *Matricaria chamomilla* white ray floret supercritical fluid extracts according to Supplementary Table S3.
