# Supplementary material for: Exploring the Potential of Supercritical Fluid Extraction of Matricaria chamomilla White Ray Florets as a Source of Bioactive (Cosmetic) Ingredients
Source: Antioxidants (Basel). 2023 May 12;12(5):1092. doi: 10.3390/antiox12051092 (PMC10215379; doi:10.3390/antiox12051092)
Supplement: Supplementary file 1 [file antioxidants-12-01092-s001.zip › Figure S1 2379695 Pastare et al.pdf]

Figure S1. GC-MS masspectra for all identified compounds in *Matricaria chamomilla* white ray floret supercritical fluid extracts according to Supplementary table S1.

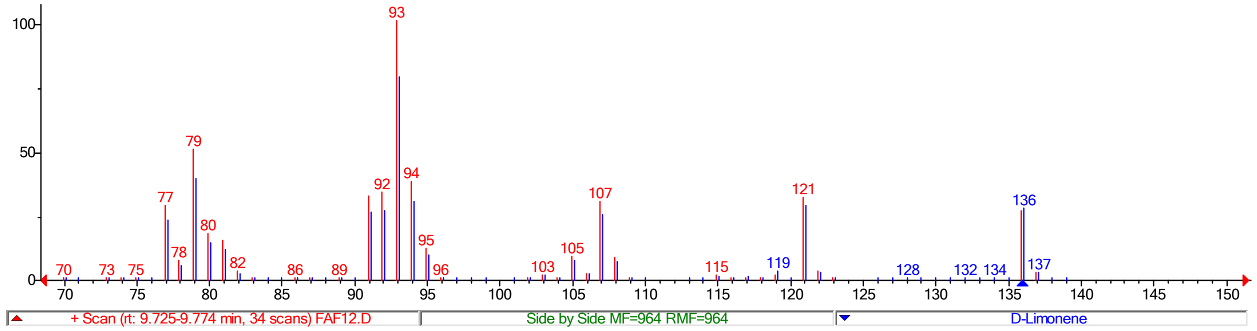

V1

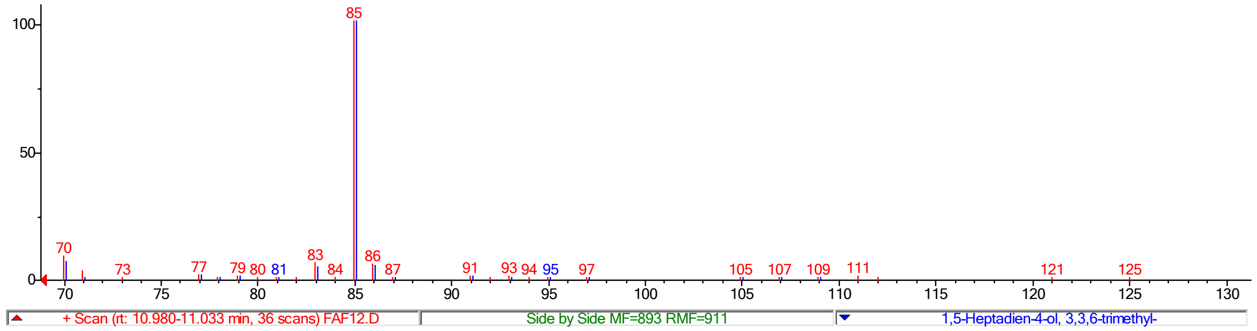

V2

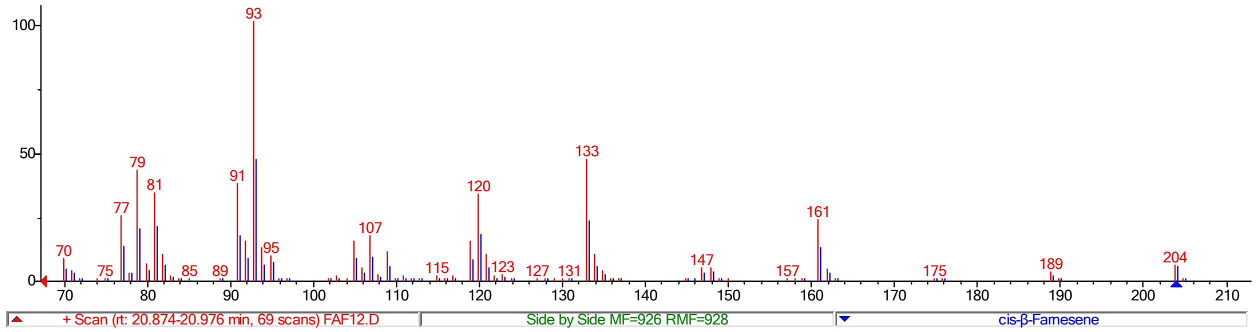

V3

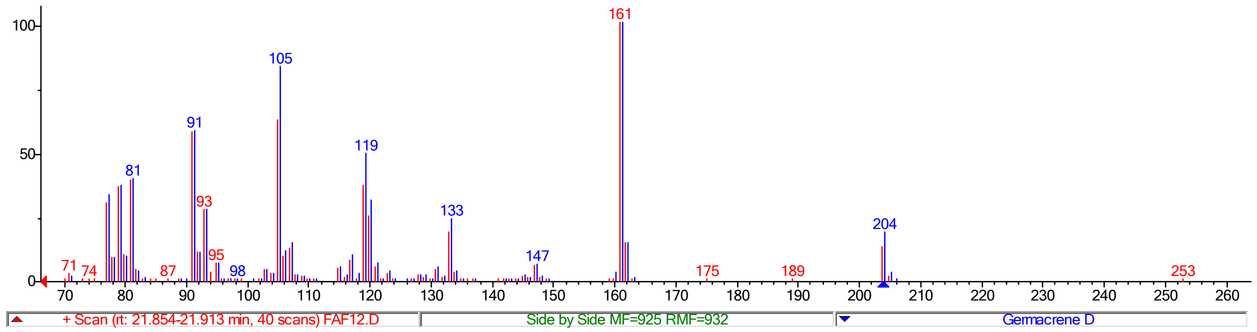

V4.

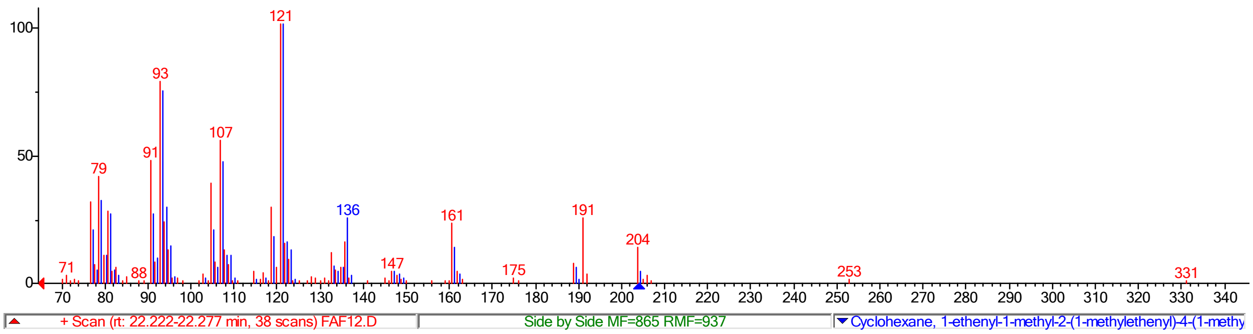
121
93
107
91
79
136
161
191
204
71
88
147
175
253
331
+ Scan (rt: 22.222-22.277 min, 38 scans) FAF12.D
Side by Side MF=865 RMF=937
Cyclohexane, 1-ethenyl-1-methyl-2-(1-methylethenyl)-4-(1-methy

V5

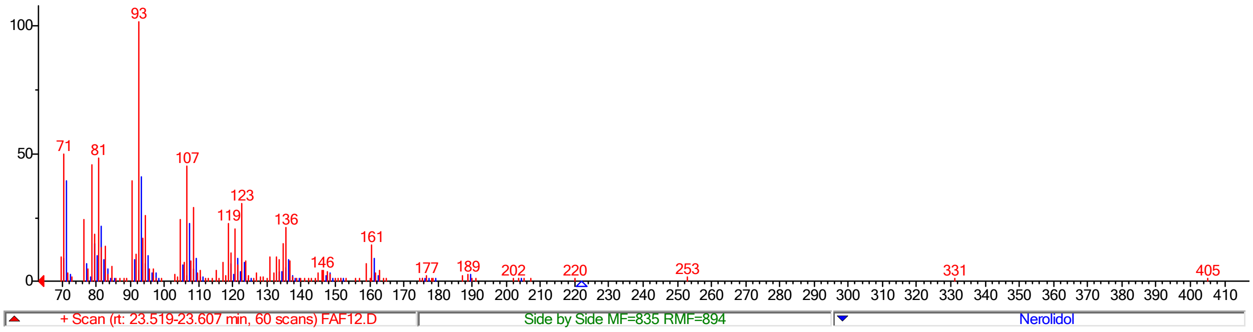
93
71
81
107
123
119
136
161
146
177
189
202
220
253
331
405
+ Scan (rt: 23.519-23.607 min, 60 scans) FAF12.D
Side by Side MF=835 RMF=894
Nerolidol

V6

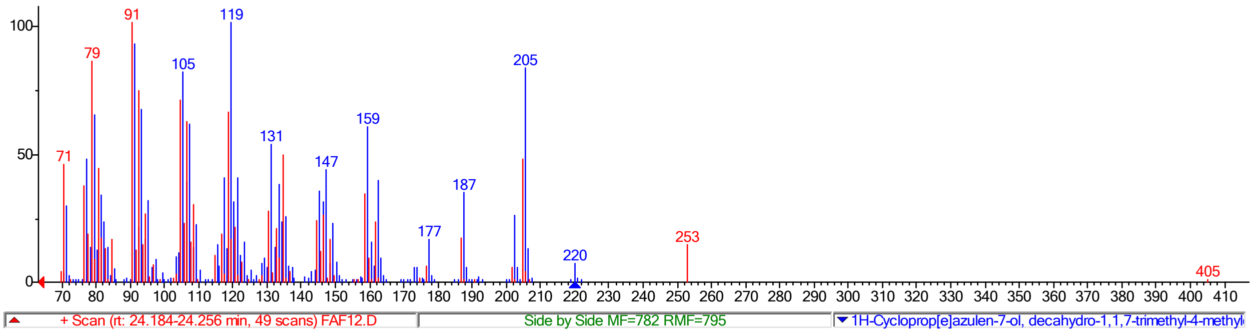
91
119
79
105
71
131
159
147
187
205
177
220
253
405
+ Scan (rt: 24.184-24.256 min, 49 scans) FAF12.D
Side by Side MF=782 RMF=795
1H-Cycloprop[e]azulen-7-ol, decahydro-1,1,7-trimethyl-4-methyl

V7.

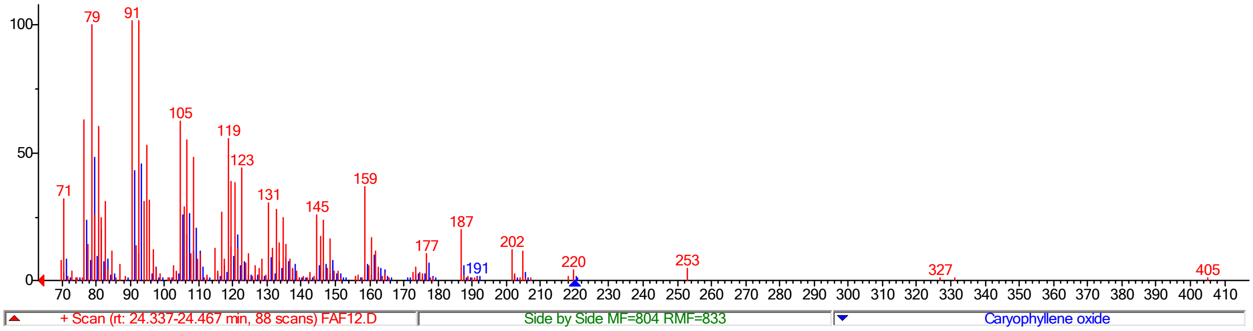
79
91
105
119
123
71
131
145
159
187
177
202
191
220
253
327
405
+ Scan (rt: 24.337-24.467 min, 88 scans) FAF12.D
Side by Side MF=804 RMF=833
Caryophyllene oxide

V8

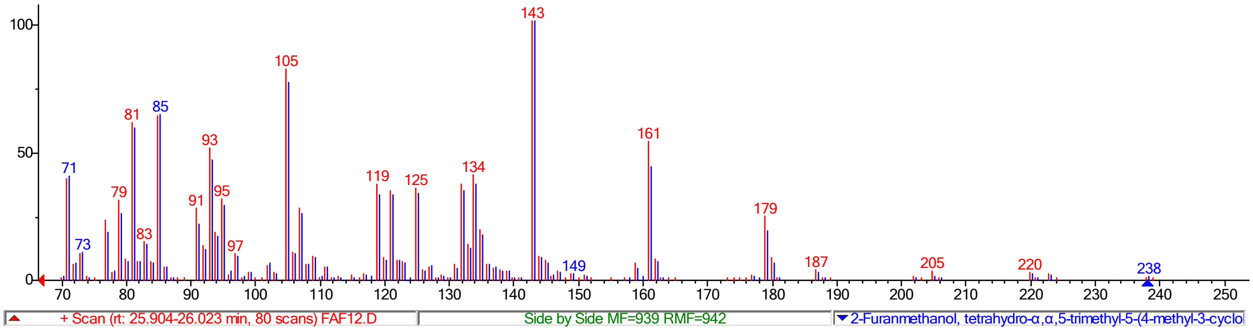
+ Scan (rt: 25.904-26.023 min, 80 scans) FAF12.D
Side by Side MF=939 RMF=942
2-Furanmethanol, tetrahydro-α,α,5-trimethyl-5-(4-methyl-3-cyclo

V9

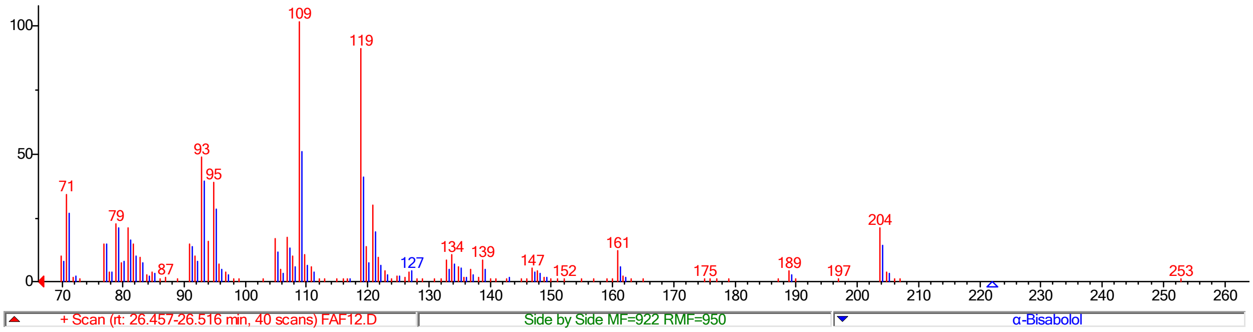
+ Scan (rt: 26.457-26.516 min, 40 scans) FAF12.D
Side by Side MF=922 RMF=950
α-Bisabolol

V10

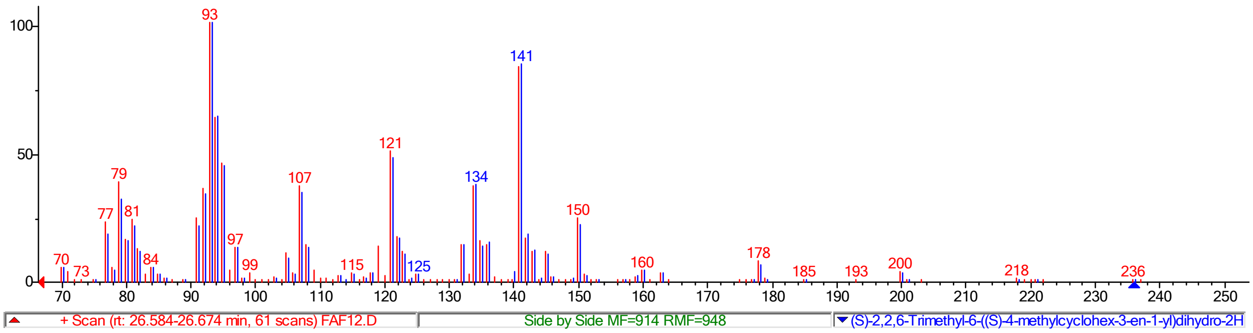
+ Scan (rt: 26.584-26.674 min, 61 scans) FAF12.D
Side by Side MF=914 RMF=948
(S)-2,2,6-Trimethyl-6-((S)-4-methylcyclohex-3-en-1-yl)dihydro-2H

V11

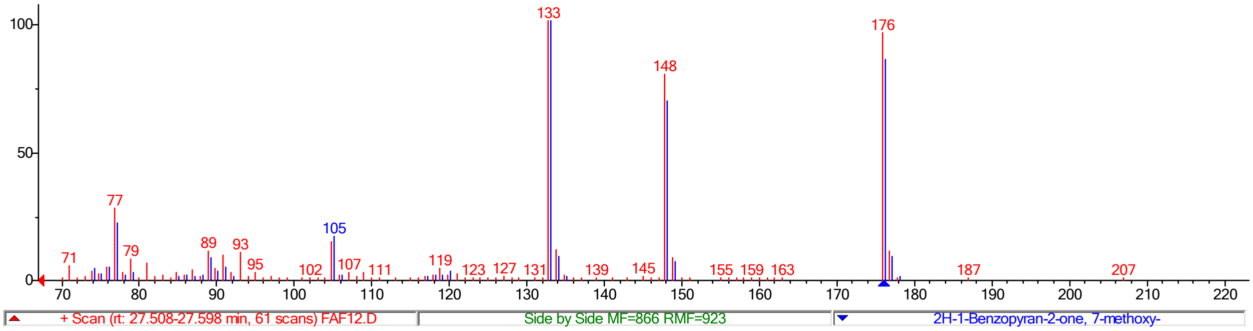
+ Scan (rt: 27.508-27.598 min, 61 scans) FAF12.D
Side by Side MF=866 RMF=923
2H-1-Benzopyran-2-one, 7-methoxy-

V12

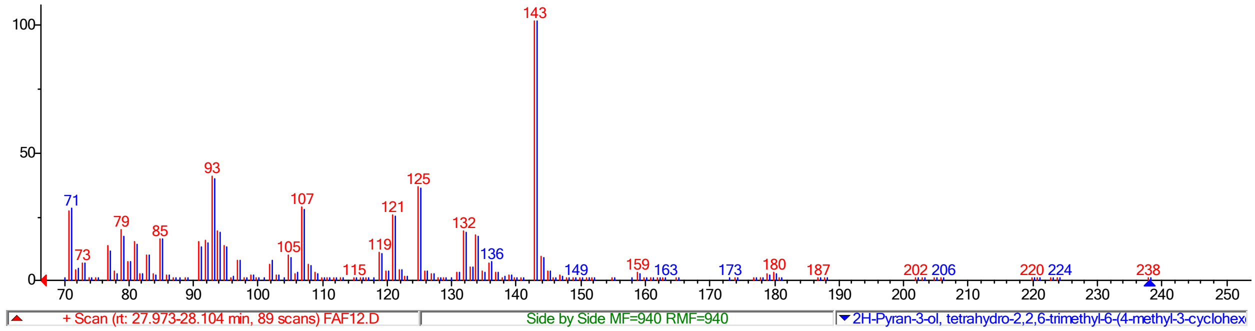

V13

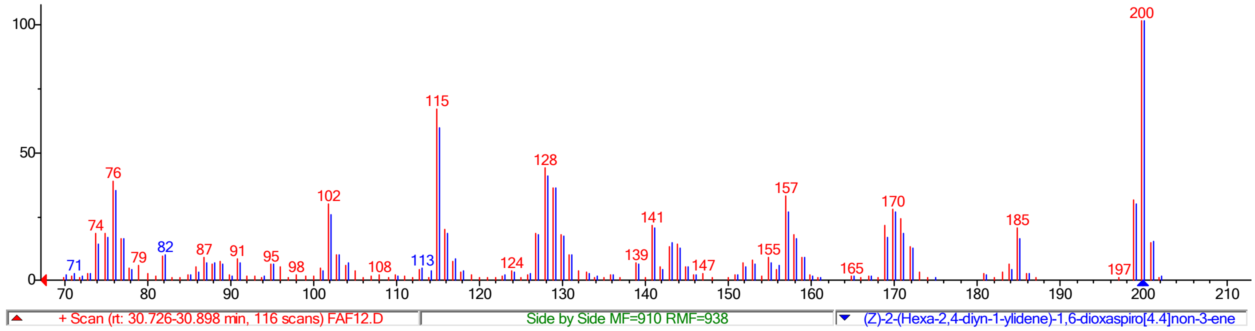

V14

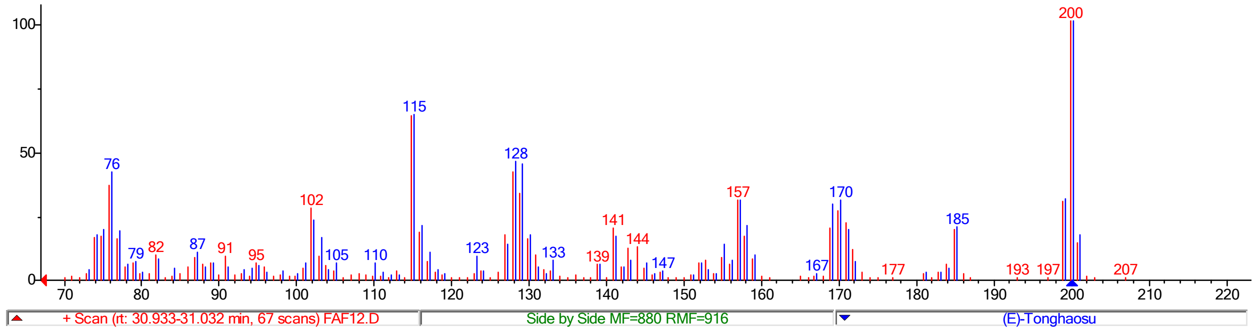

V15

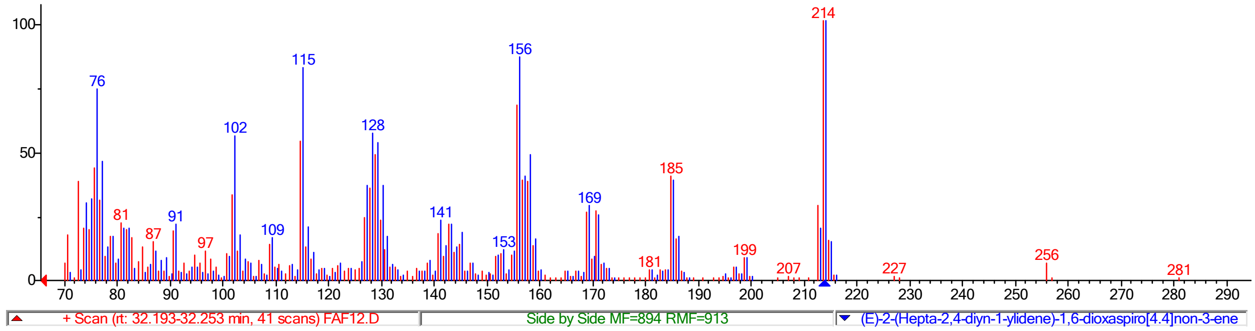

V16

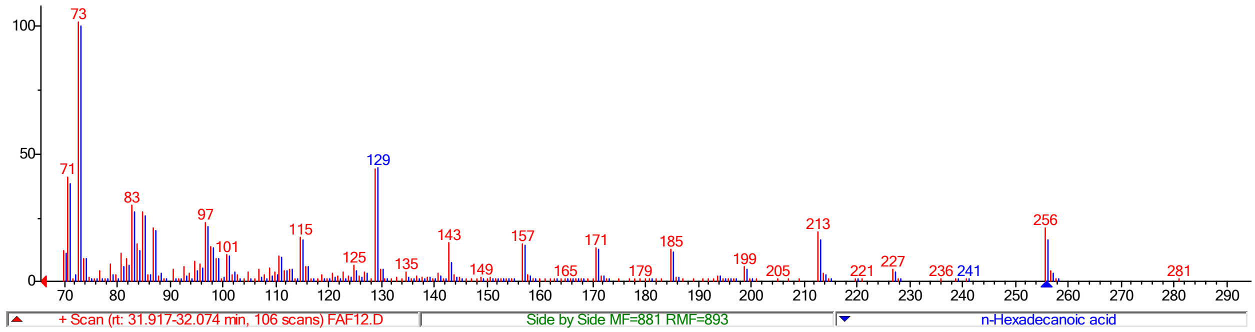

V17

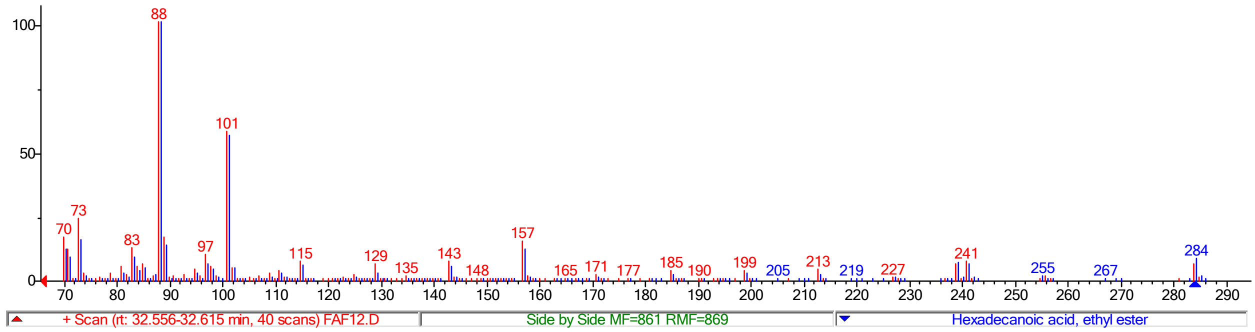

V18

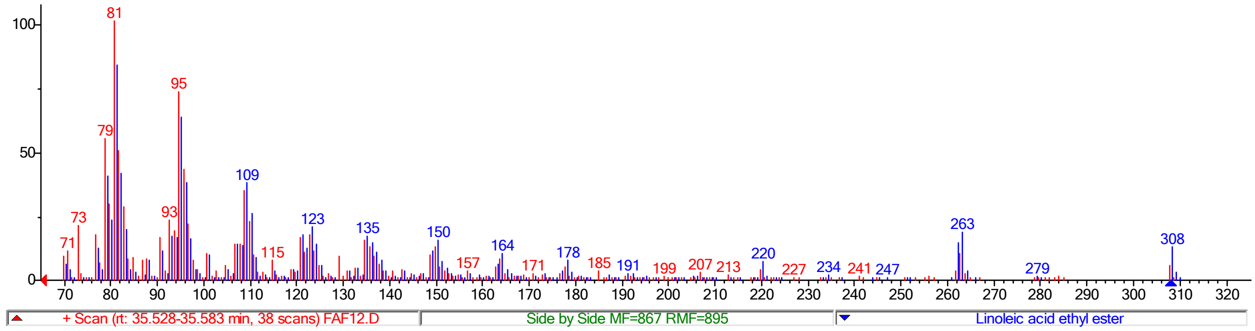

V19

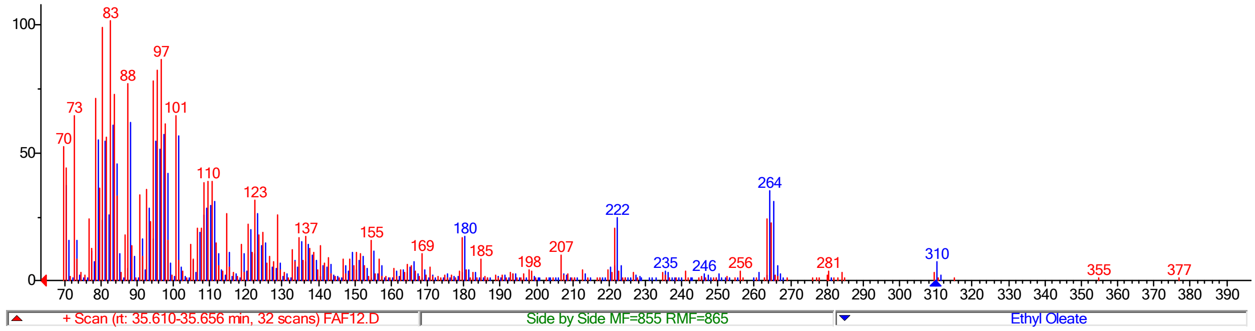

V20

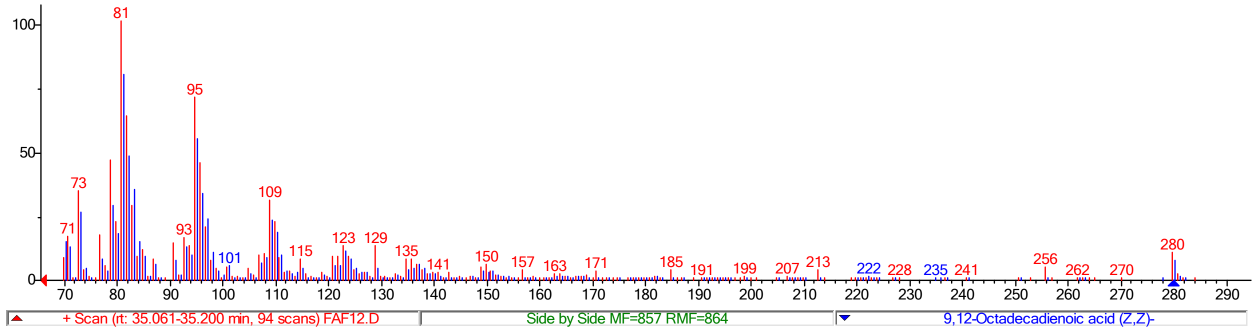

V21

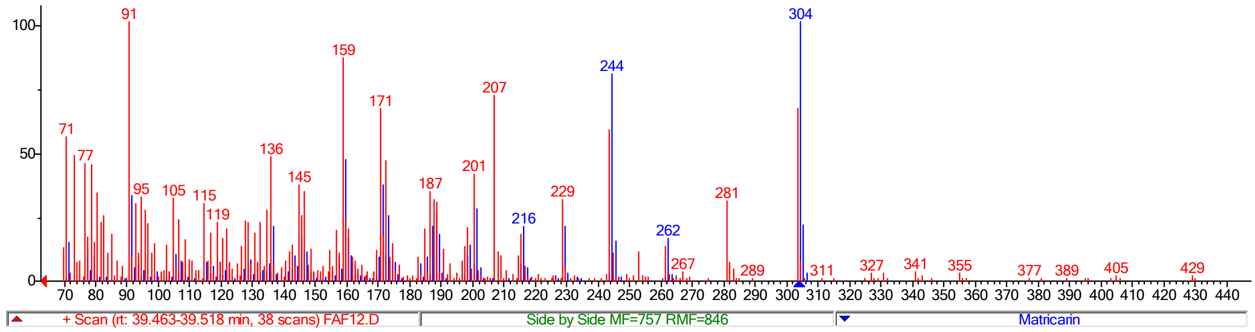

V22

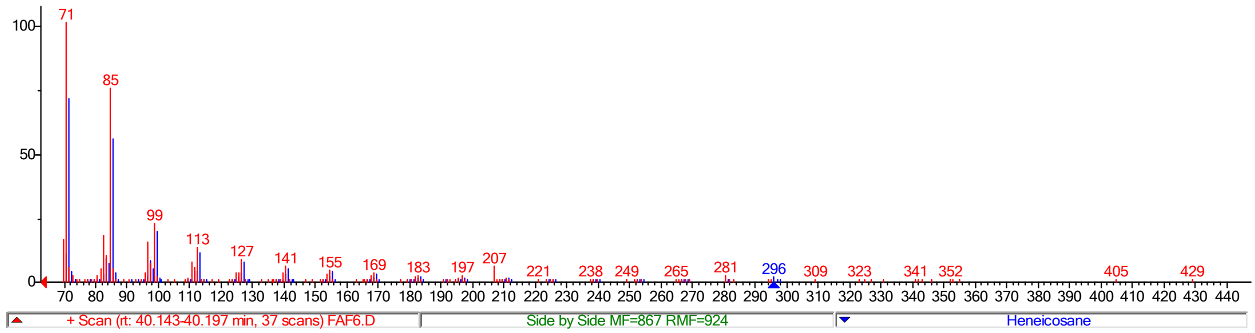

V23

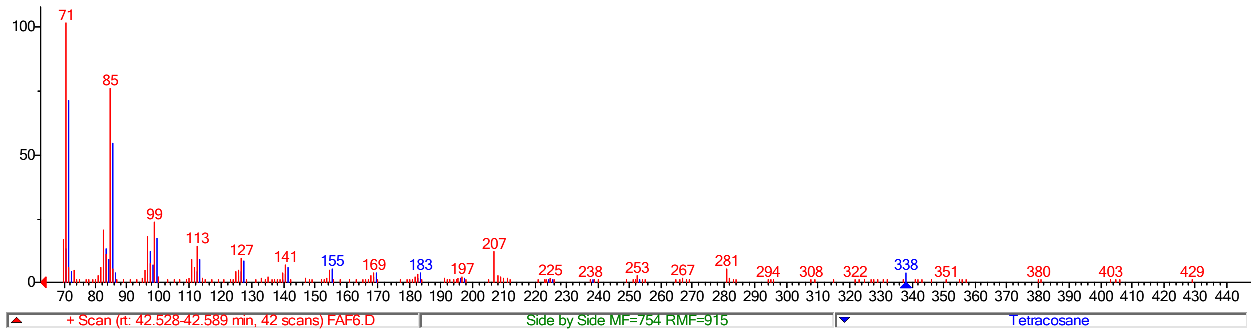

V24

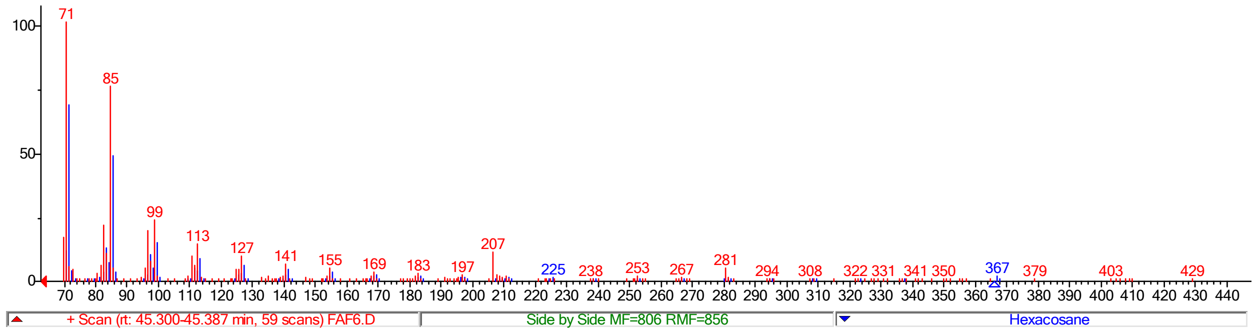

V25

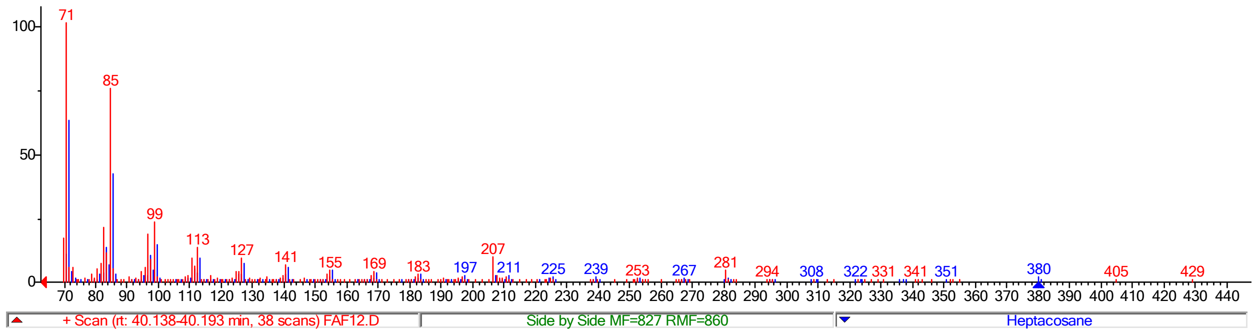

V26

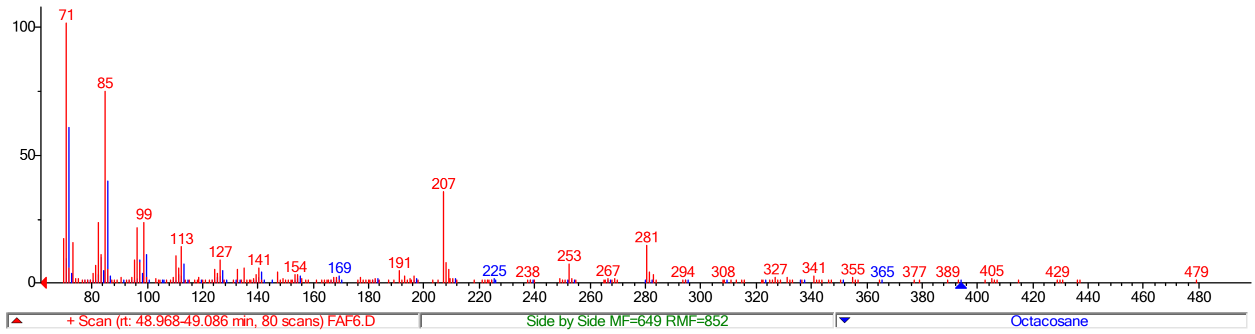

V27

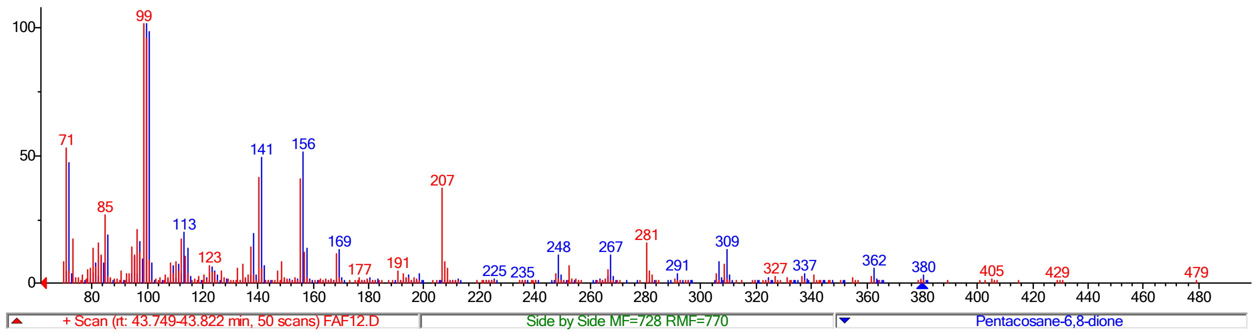

V28

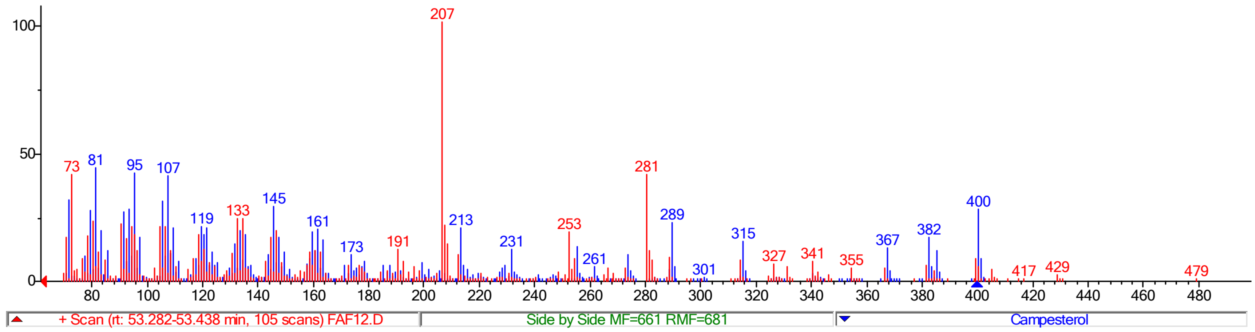

V29

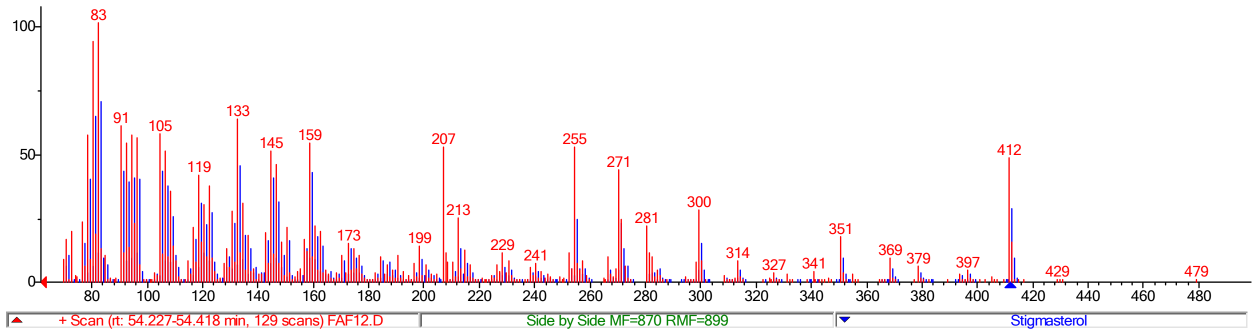

V30

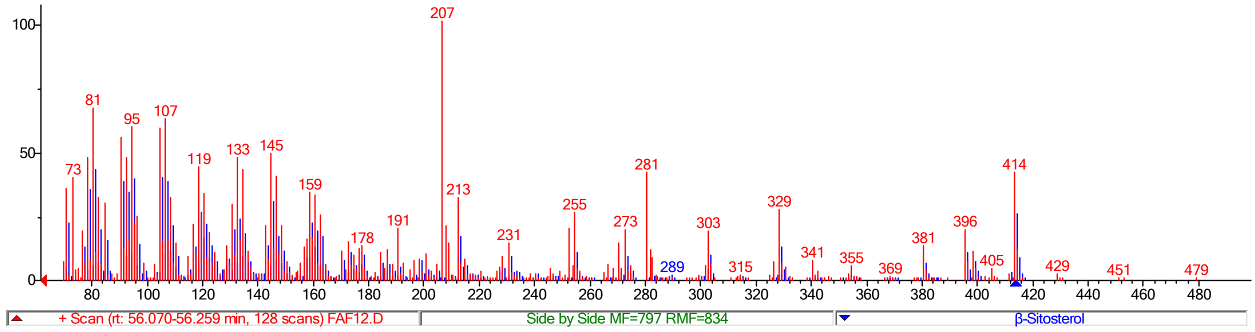

V31

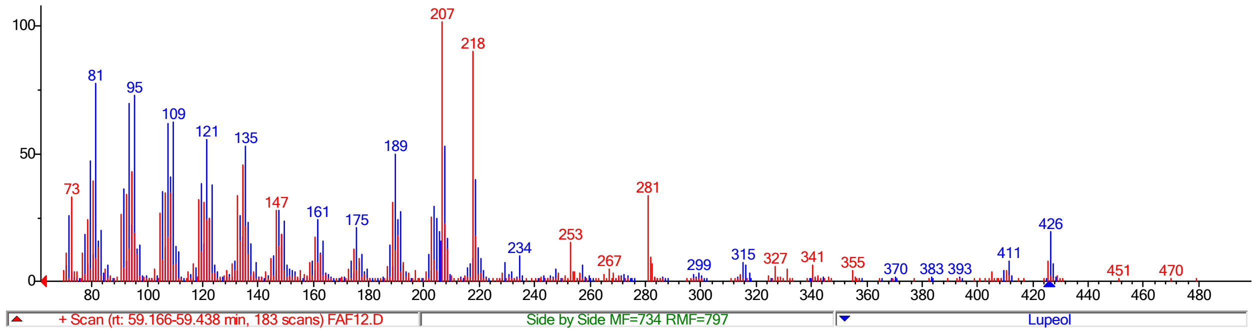

V32

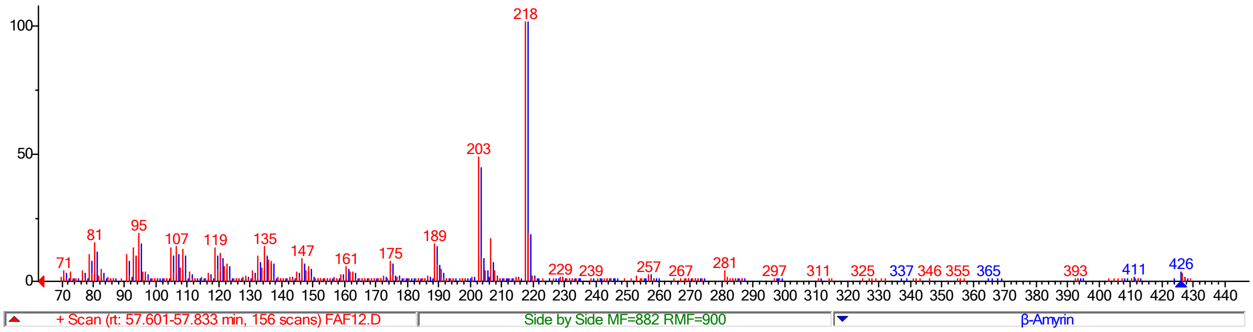

V33

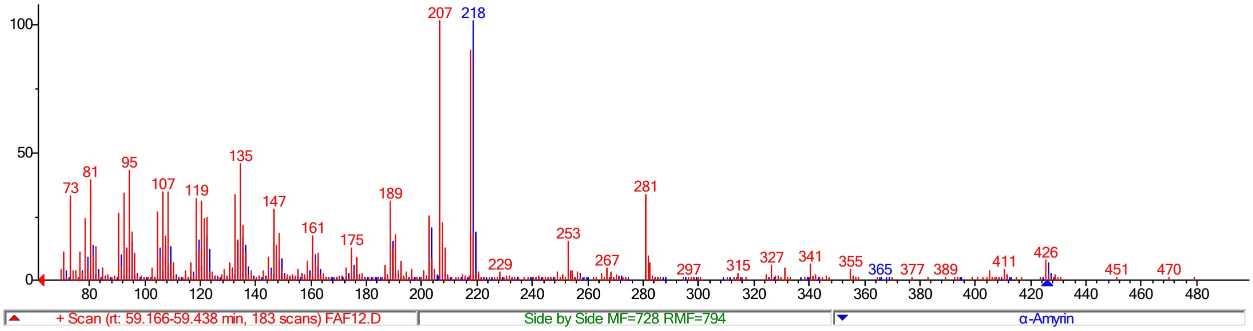

V34

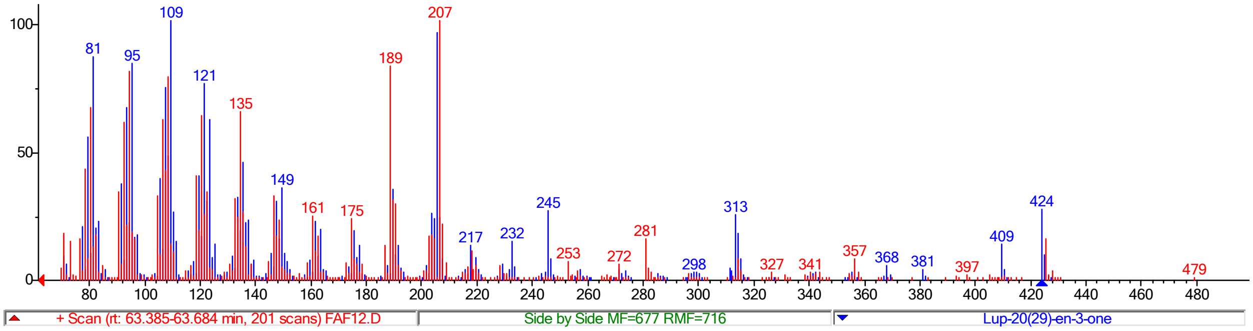

V35

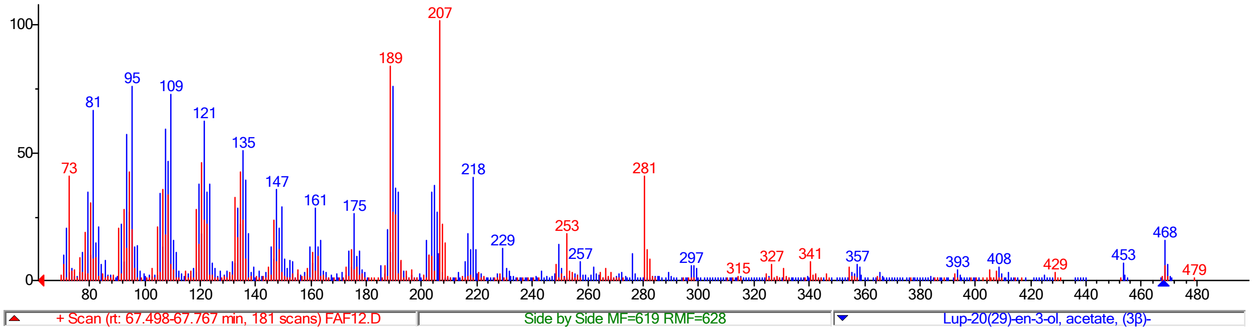

V36

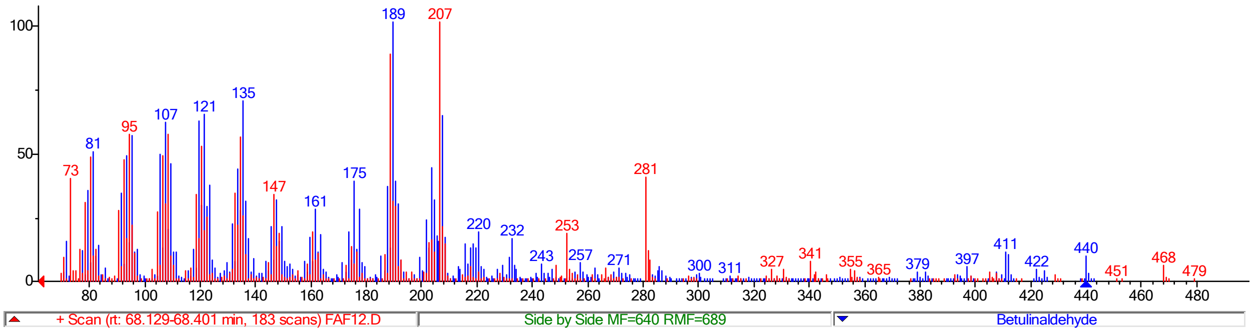

100
50
0
73
81
95
107
121
135
147
161
175
189
207
220
232
243
253
257
271
281
300
311
327
341
355
365
379
397
411
422
440
451
468
479
80
100
120
140
160
180
200
220
240
260
280
300
320
340
360
380
400
420
440
460
480
+ Scan (rt: 68.129-68.401 min, 183 scans) FAF12.D
Side by Side MF=640 RMF=689
Betulinaldehyde

V37
