# Supplementary material for: Exploring the Potential of Supercritical Fluid Extraction of Matricaria chamomilla White Ray Florets as a Source of Bioactive (Cosmetic) Ingredients
Source: Antioxidants (Basel). 2023 May 12;12(5):1092. doi: 10.3390/antiox12051092 (PMC10215379; doi:10.3390/antiox12051092)
Supplement: Supplementary file 1 [file antioxidants-12-01092-s001.zip › Figure S2 2379695 Pastare et al.pdf]

Figure S2. LC-HRMS masspectra for identified compounds in *Matricaria chamomilla* white ray floret supercritical fluid extracts according to Supplementary Table S3.

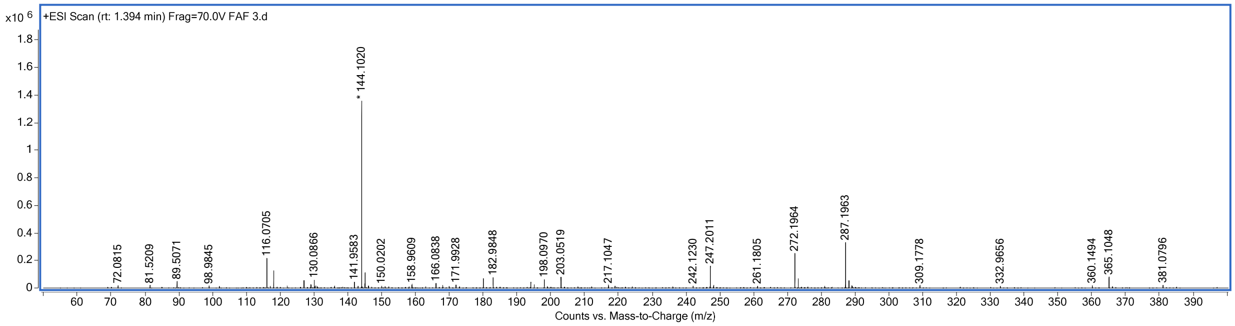

AA1

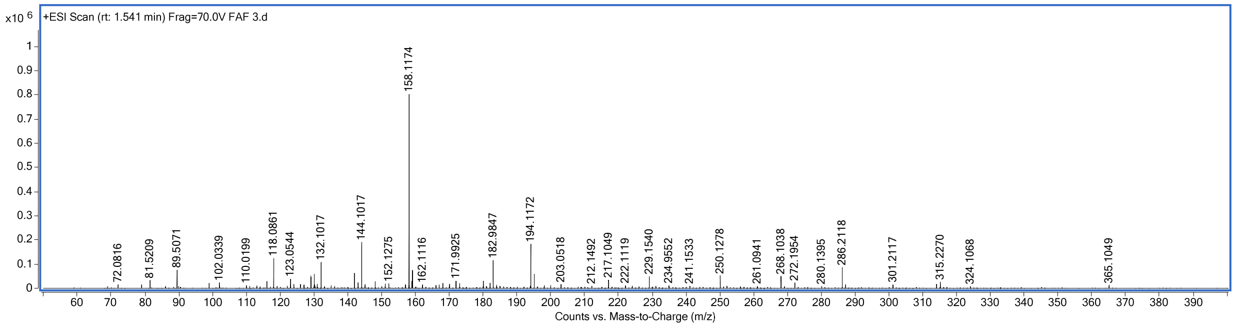

AA2

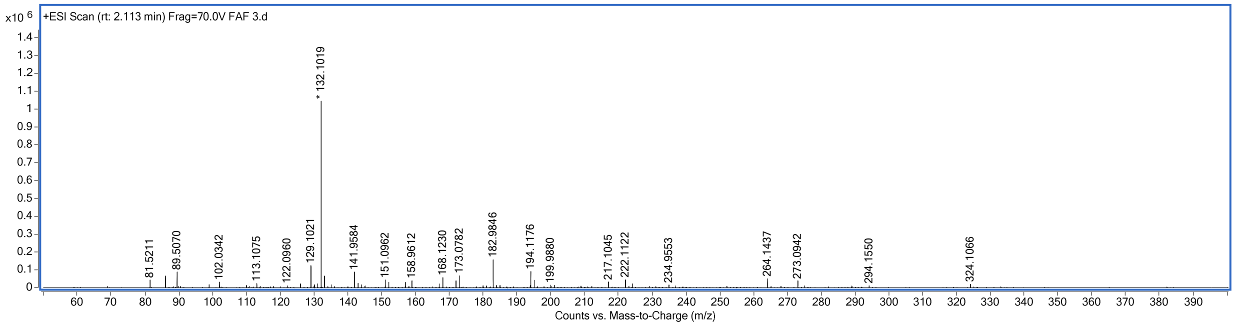

AA3

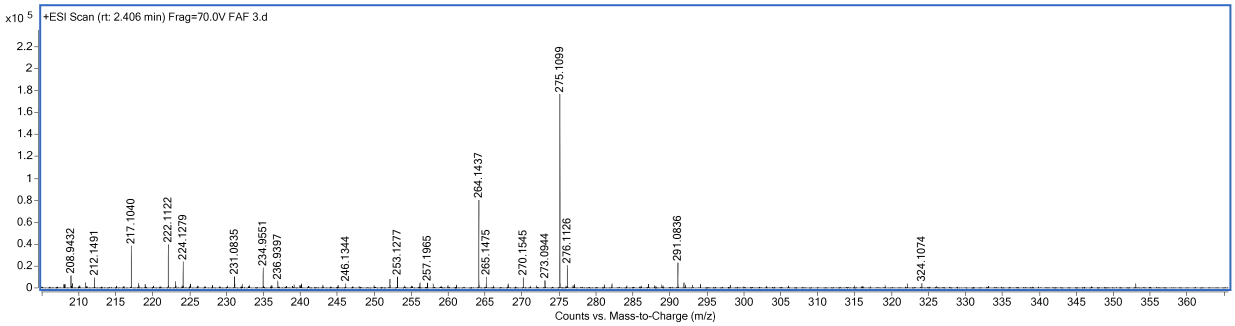

AA4

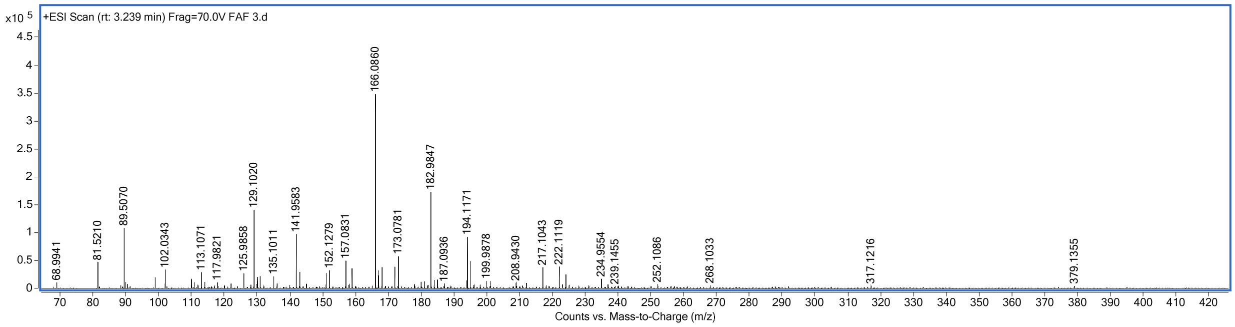

AA5

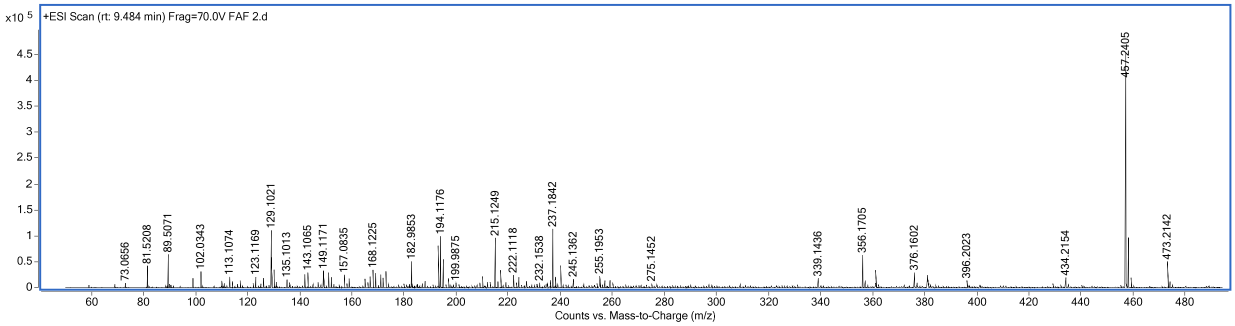

AA6

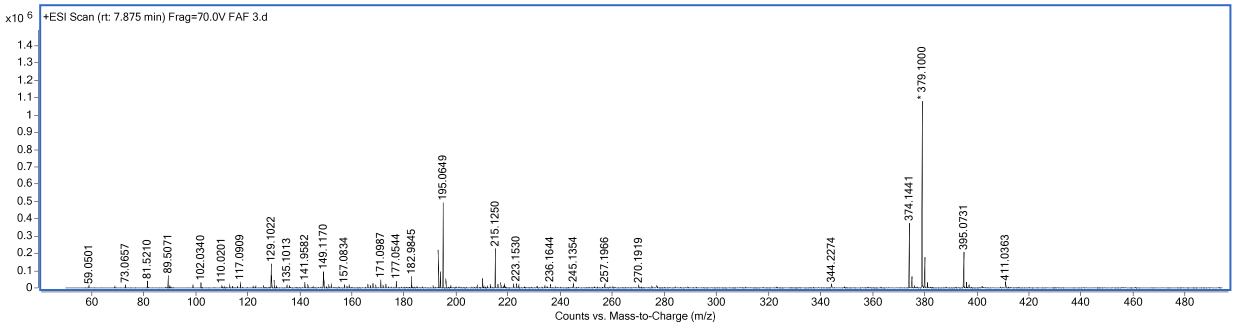

FA1

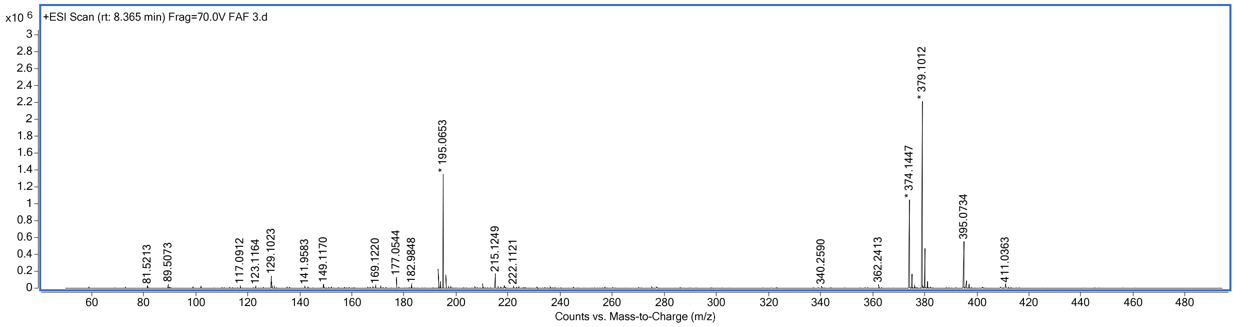

FA2

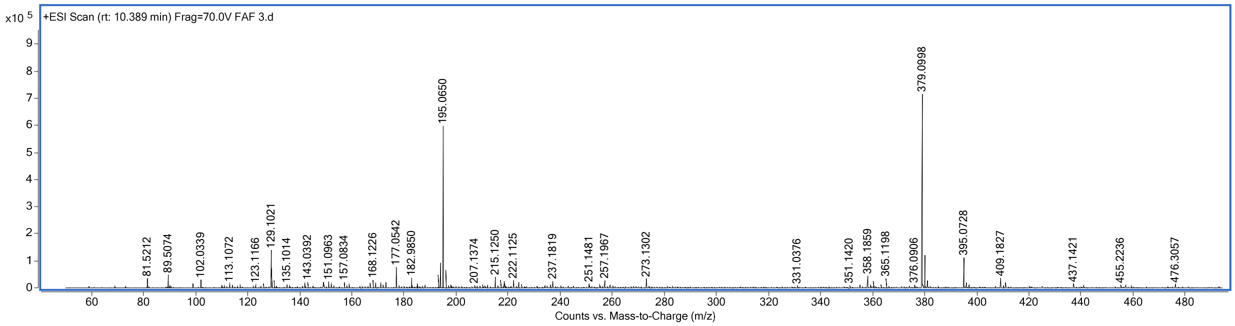

FA3

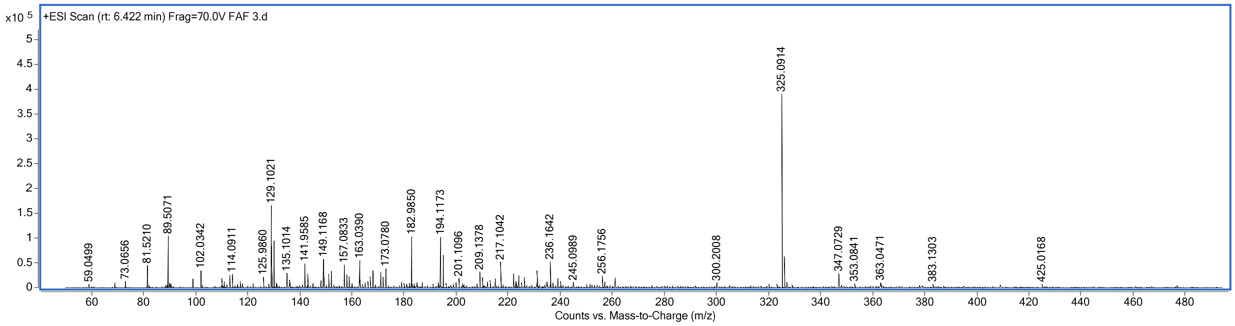

COU1

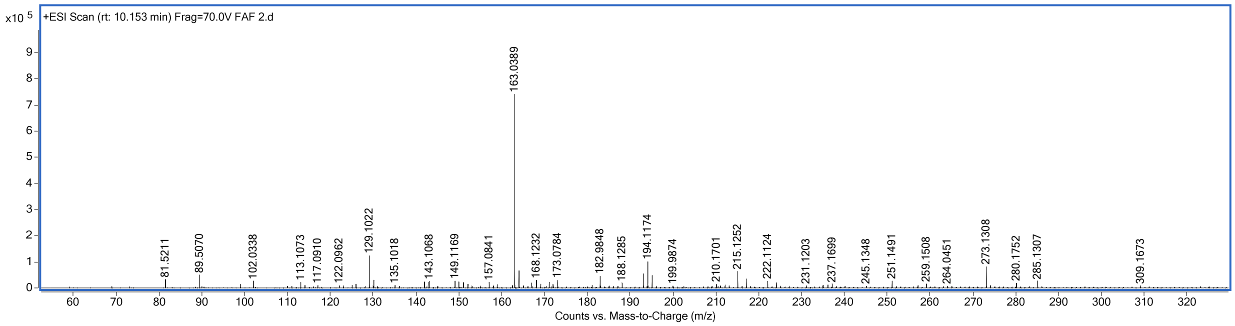

COU2

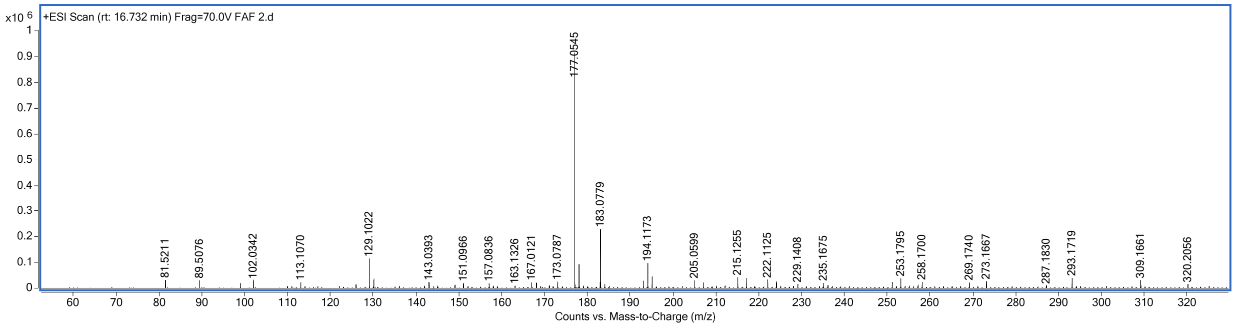

COU3

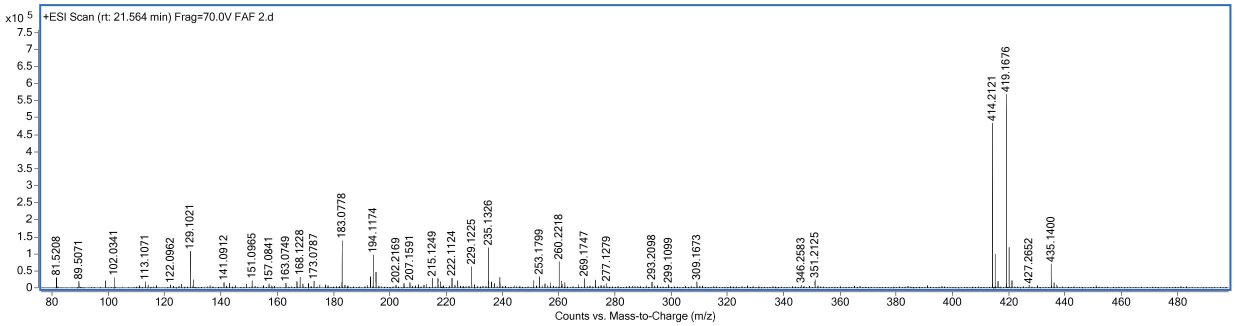

COU4

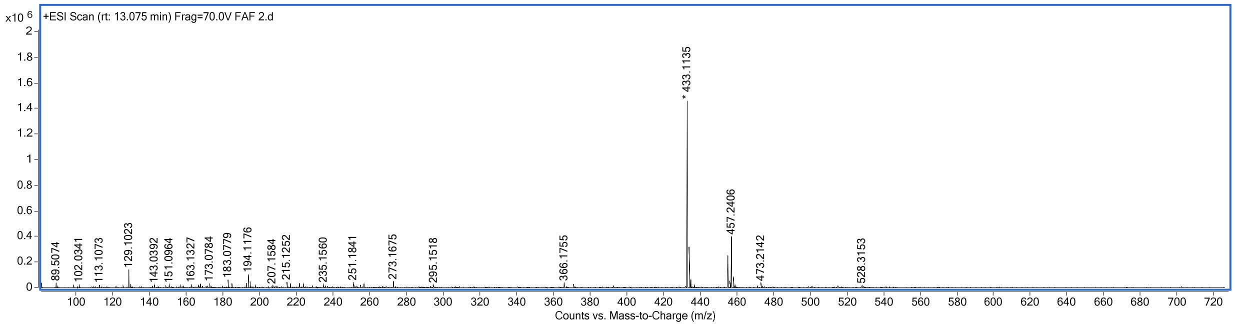

API1

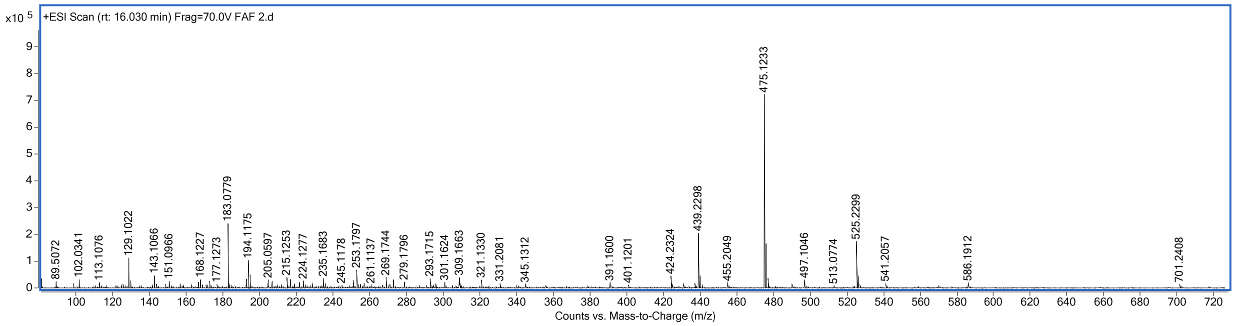

API2

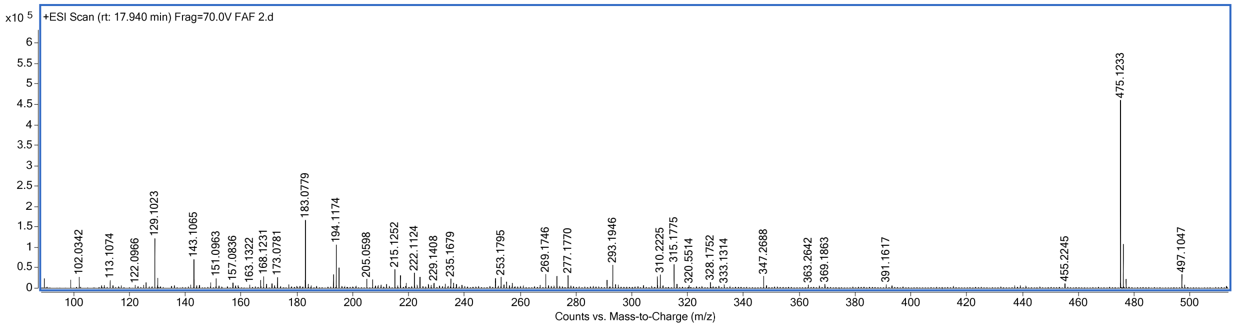

API3

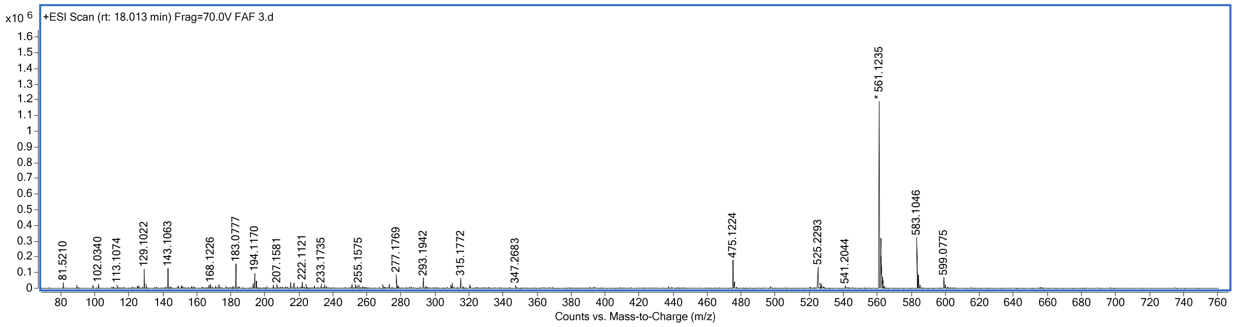

API4

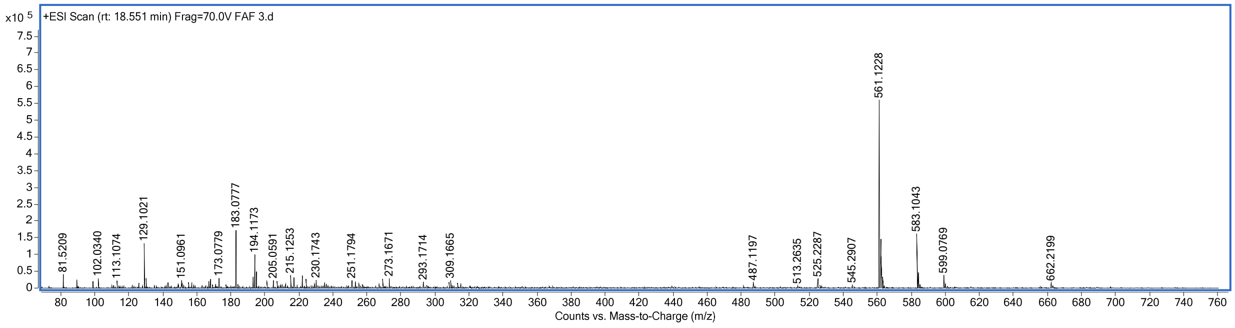

API5

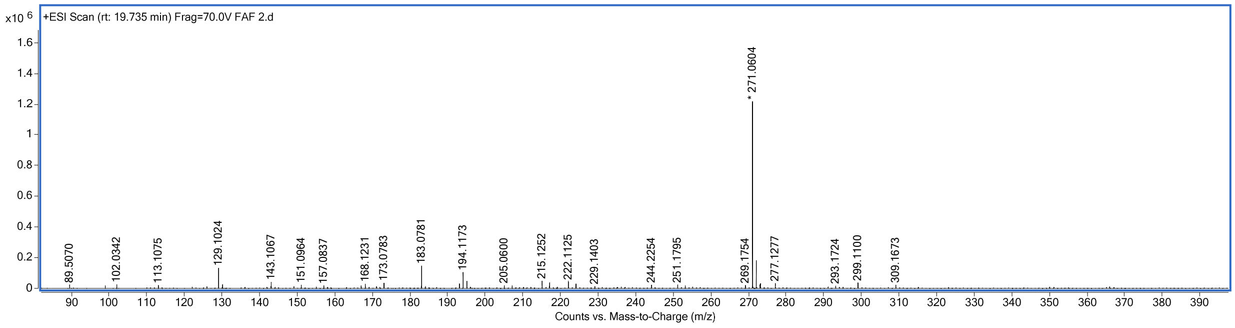

API6
