# Supplementary material for: Exploring the Potential of Supercritical Fluid Extraction of Matricaria chamomilla White Ray Florets as a Source of Bioactive (Cosmetic) Ingredients
Source: Antioxidants (Basel). 2023 May 12;12(5):1092. doi: 10.3390/antiox12051092 (PMC10215379; doi:10.3390/antiox12051092)
Supplement: Supplementary file 1 [file antioxidants-12-01092-s001.zip › Table S1 2379695 Pastare et al.pdf]

**Table S1.** Chemical composition of separated volatile compounds from *Matricaria chamomilla* white ray floret supercritical fluid extracts.

| V* | Compound [b] | RI [a] | Amount mg $mL^{-1}$ α-bisabolol equivalents | | | | | | | | | | | | | Class |
|---|---|---|---|---|---|---|---|---|---|---|---|---|---|---|---|---|
| | | | FAF1 | FAF 2 | FAF 3 | FAF4 | FAF5 | FAF6 | FAF7 | FAF8 | FAF 9 | FAF10 | FAF11 | FAF12 | FAF13 | |
| 1 | Limonene | 1030 | 0.20±0.02 | - | 0.40±0.03 | 0.02±0.00 | 0.12±0.02 | 0.37±0.04 | 0.29±0.03 | 0.15±0.01 | 0.05±0.01 | 0.25±0.02 | 0.26±0.02 | 0.41±0.02 | 0.17±0.02 | Monoterpenes |
| 2 | Artemisia alcohol | 1084 | 0.01±0.00 | - | 0.01±0.00 | 0.01±0.00 | 0.01±0.00 | 0.02±0.01 | 0.01±0.00 | - | - | - | 0.01±0.00 | - | - | Carboxylic acid esters |
| 3 | (E)-β-Farnesene | 1444 | 1.13±0.11 | - | 0.13±0.02 | - | 0.38±0.03 | 1.78±0.13 | 1.30±0.21 | 1.44±0.09 | 0.15±0.02 | 1.90±0.21 | 1.61±0.17 | 3.64±0.54 | 1.38±0.23 | Sesquiterpenoids |
| 4 | Germacrene D | 1481 | 0.10±0.01 | - | 0.01±0.00 | - | 0.02±0.01 | 0.13±0.01 | 0.09±0.01 | 0.16±0.01 | 0.01±0.00 | 0.14±0.01 | 0.15±0.01 | 0.29±0.01 | 0.09±0.02 | Sesquiterpenoids |
| 5 | Elixene | 1485 | 0.05±0.01 | - | 0.01±0.00 | 0.01±0.00 | - | 0.05±0.01 | 0.04±0.01 | 0.09±0.01 | - | - | 0.07±0.01 | - | - | Sesquiterpenoids |
| 6 | E-Nerolidol | 1564 | - | - | - | - | - | - | - | - | - | - | - | 0.16±0.02 | - | Sesquiterpenoids |
| 7 | Spathulenol | 1576 | 0.06±0.01 | - | - | - | 0.01±0.00 | 0.10±0.01 | 0.06±0.01 | 0.06±0.01 | 0.01±0.00 | 0.63±0.04 | 0.08±0.01 | 2.21±0.18 | 0.30±0.02 | Sesquiterpenoids |
| 8 | Caryophyllene oxide | 1581 | - | - | - | - | - | - | - | - | - | - | - | 0.14±0.02 | - | Sesquiterpenoids |
| 9 | α-Bisabolol oxide B | 1655 | 0.28±0.03 | 0.10±0.01 | 0.01±0.00 | - | 0.06±0.01 | 0.41±0.03 | 0.27±0.02 | 0.34±0.02 | 0.04±0.01 | 2.06±0.15 | 0.38±0.02 | 5.69±0.84 | 1.05±0.06 | Tetrahydrofurans |
| 10 | α-Bisabolol | 1668 | - | - | - | - | - | - | - | - | - | 0.08±0.01 | - | 0.32±0.02 | 0.04±0.01 | Sesquiterpenoids |
| 11 | α-Bisabolone oxide A | 1679 | 0.22±0.02 | 0.06±0.00 | - | - | 0.04±0.01 | 0.30±0.02 | 0.20±0.02 | 0.30±0.02 | 0.03±0.00 | 1.21±0.12 | 0.30±0.02 | 3.26±0.66 | 0.61±0.08 | Tetrahydrofurans |
| 12 | Herniarin | 1732 | - | - | - | - | - | - | - | - | - | - | - | 0.22±0.02 | - | Coumarins |
| 13 | α-Bisabolol oxide A | 1744 | 0.24±0.03 | 0.12±0.02 | 0.01±0.00 | - | 0.05±0.01 | 0.44±0.03 | 0.21±0.02 | 0.20±0.01 | 0.04±0.01 | 2.25±0.34 | 0.32±0.01 | 6.22±0.72 | 1.22±0.14 | Tetrahydrofurans |
| 14 | cis-ene-yne-Dicycloether | 1849 | 0.37±0.05 | 0.21±0.03 | 0.01±0.00 | - | 0.06±0.01 | 0.67±0.03 | 0.33±0.01 | 0.28±0.01 | 0.02±0.00 | 3.13±0.65 | 0.48±0.02 | 9.02±1.02 | 1.65±0.07 | Spiroethers |
| 15 | E-En-yn-dicycloether | 1902 | 0.07±0.01 | 0.05±0.01 | - | - | 0.04±0.01 | 0.13±0.02 | 0.06±0.01 | 0.05±0.01 | 0.01±0.00 | 0.69±0.18 | 0.09±0.01 | 2.40±0.09 | 0.39±0.02 | Spiroethers |
| 16 | (E)-Tibetin spiroether | 1961 | - | - | - | - | - | - | - | - | - | 0.09±0.01 | - | 0.31±0.02 | 0.05±0.01 | Spiroethers |
| 17 | Palmitic acid | 1968 | - | - | - | - | - | - | - | - | - | - | - | 0.83±0.05 | - | Fatty acids |
| 18 | Ethyl palmitate | 1993 | - | - | - | - | - | - | - | - | - | 0.08±0.01 | - | 0.25±0.01 | 0.06±0.01 | Fatty acid esters |
| 19 | Ethyl linoleate | 2162 | 0.01±0.00 | - | - | - | - | 0.02±0.01 | - | - | - | 0.16±0.01 | 0.01±0.00 | 0.39±0.02 | 0.11±0.02 | Fatty acid esters |
| 20 | Ethyl oleate | 2173 | - | - | - | - | - | - | - | - | - | 0.13±0.01 | - | 0.23±0.03 | 0.08±0.01 | Fatty acid esters |
| 21 | Linoleic acid | 2133 | - | - | - | - | - | - | - | - | - | 0.12±0.01 | - | 1.54±0.14 | 0.07±0.01 | Fatty acids |
| 22 | Matricarin | 2310 | - | - | - | - | - | - | - | - | - | - | - | 0.14±0.02 | - | Lactones |
| 23 | Heineicosane | 2100 | 0.01±0.00 | - | - | - | - | 0.04±0.01 | 0.01±0.00 | - | - | 0.09±0.01 | 0.02±0.01 | 0.13±0.01 | 0.08±0.01 | Hydrocarbons |
| 24 | Tetracosane | 2400 | 0.28±0.02 | 0.14±0.02 | 0.01±0.00 | 0.01±0.00 | 0.09±0.01 | 0.54±0.09 | 0.24±0.02 | 0.18±0.02 | - | 0.24±0.02 | 0.36±0.03 | 0.32±0.02 | 0.22±0.01 | Hydrocarbons |
| 25 | Hexacosane | 2600 | 1.19±0.18 | - | 0.04±0.00 | 0.01±0.00 | 0.09±0.01 | 1.17±0.11 | 0.66±0.04 | 2.92±0.16 | - | - | 2.05±0.38 | - | - | Hydrocarbons |
| 26 | Heptacosane | 2700 | 0.51±0.06 | 0.05±0.01 | 0.01±0.00 | 0.01±0.00 | 0.08±0.01 | 0.69±0.03 | 0.33±0.01 | 0.96±0.08 | - | 0.09±0.02 | 0.83±0.20 | - | 0.08±0.01 | Hydrocarbons |
| 27 | Octacosane | 2800 | 0.71±0.02 | - | - | - | 0.04±0.01 | 0.48±0.07 | 0.28±0.01 | 2.07±0.45 | - | - | 1.28±0.14 | - | - | Hydrocarbons |
| 28 | Pentacosane-6,8-dione | 2781 | 0.05±0.01 | - | - | - | - | 0.05±0.01 | 0.02±0.00 | 0.12±0.02 | - | 0.11±0.01 | 0.09±0.00 | 0.47±0.2 | 0.11±0.01 | Beta-diketones |
| 29 | Campesterol | 3131 | - | - | - | - | - | - | - | - | - | - | - | 0.27±0.01 | - | Phytosterol |
| 30 | Stigmasterol | 3170 | 0.17±0.02 | 0.37±0.01 | 0.04±0.00 | 0.05±0.01 | - | 0.10±0.00 | 0.05±0.01 | 0.15±0.01 | 0.18±0.02 | 0.71±0.05 | 0.13±0.01 | 3.05±0.26 | 0.63±0.05 | Phytosterol |
| 31 | β-Sitosterol | 3200 | 0.06±0.01 | 0.23±0.01 | - | 0.05±0.01 | - | - | - | - | 0.09±0.01 | 0.32±0.02 | - | 1.15±0.08 | 0.30±0.04 | Phytosterol |
| 32 | Lupeol | 3270 | 0.63±0.05 | 1.61±0.13 | 0.09±0.01 | 0.29±0.02 | 0.32±0.02 | 0.44±0.03 | 0.35±0.02 | 0.10±0.01 | 3.89±0.26 | 3.52±0.22 | 0.27±0.02 | 4.93±0.41 | 3.21±0.52 | Triterpenes |
| 33 | β-Amyrin | 3337 | 0.04±0.00 | 0.09±0.01 | - | - | - | - | - | 0.06±0.01 | 0.59±0.13 | 0.39±0.03 | 0.03±0.00 | 1.16± | 0.36±0.01 | Triterpenes |
| 34 | α-Amyrin | 3376 | 0.21±0.01 | 0.48±0.02 | - | 0.05±0.01 | 0.14±0.02 | 0.28±0.01 | - | 0.06±0.01 | 3.51±0.56 | 1.94±0.34 | 0.17±0.01 | 4.79±0.23 | 1.83±0.26 | Triterpenes |
| 35 | Lupenone | 3384 | 1.13±0.07 | 3.46±0.32 | 0.16±0.02 | 0.63±0.05 | 0.40±0.04 | 0.56±0.03 | 0.50±0.03 | - | 4.35±0.32 | 3.78±0.44 | 0.28±0.02 | 4.75±0.31 | 3.59±0.37 | Triterpenes |
| 36 | Lupeol acetate | 3525 | 0.04±0.00 | 0.05±0.01 | - | - | - | 0.07±0.01 | 0.02±0.00 | - | 0.05±0.00 | 0.28±0.02 | 0.04±0.00 | 0.74±0.12 | 0.24±0.02 | Triterpenes |
| 37 | Betulin aldehyde | 3629 | 0.07±0.01 | 0.10±0.01 | - | - | - | 0.12±0.02 | 0.06±0.01 | - | 0.08±0.01 | 0.41±0.03 | 0.06±0.01 | 1.09±0.25 | 0.38±0.01 | Triterpenes |

*V – Volatile organic compounds. [a]Retention indexes (RI) determined on the HP-5MS capillary column. [b]Based on NIST (National Institute of Standards and Technology) MS Search 2.2 library. A blank indicates that the compound has not been detected.
