# Supplementary material for: Exploring the Potential of Supercritical Fluid Extraction of Matricaria chamomilla White Ray Florets as a Source of Bioactive (Cosmetic) Ingredients
Source: Antioxidants (Basel). 2023 May 12;12(5):1092. doi: 10.3390/antiox12051092 (PMC10215379; doi:10.3390/antiox12051092)
Supplement: Supplementary file 1 [file antioxidants-12-01092-s001.zip › Table S3 2379695 Pastare et al.pdf]

Table S3. LC-qTOF-MS data of selected nonvolatile compounds detected in *Matricaria chamomilla* white ray floret supercritical fluid extracts in positive ionization mode.

	**Extract**	**FAF1**	**FAF2**	**FAF3**	**FAF4**	**FAF5**	**FAF6**	**FAF7**	**FAF8**	**FAF9**	**FAF10**	**FAF11**	**FAF12**	**FAF13**
**Number**	**Proposed compound**	**Amino acids[a], μg mL^{-1}**												
AA1	Proline betaine	0.75±0.15	2.70±0.31	2.35±0.33	2.87±0.18	1.61±0.24	0.16±0.01	0.13±0.01	-	0.06±0.00	0.40±0.03	0.08±0.01	0.42±0.02	0.37±0.02
AA2	(S)-Homostachydrine	0.62±0.08	2.38±0.28	0.16±0.01	2.30±0.25	0.74±0.16	0.05±0.00	0.05±0.00	-	-	0.01±0.00	0.03±0.00	0.16±0.01	0.13±0.01
AA3	Leucine/Isoleucine	1.09±0.09	4.16±0.36	1.91±0.05	3.36±0.27	0.62±0.11	0.10±0.00	0.12±0.01	-	-	0.20±0.01	0.05±0.00	0.13±0.01	0.22±0.01
AA4	Prolyl-Histidine	0.22±0.01	0.88±0.14	0.38±0.02	0.82±0.12	-	-	-	-	-	-	-	-	-
AA5	Phenylalanine	0.35±0.02	0.72±0.06	0.37±0.02	0.67±0.11	0.17±0.01	0.38±0.02	0.01±0.00	0.31±0.02	-	0.71±0.04	0.34±0.02	2.31±0.17	0.70±0.13
AA6	H-DL-Leu-DL-Met-DL-Pro-DL-Pro-OH	0.15±0.01	0.59±0.02	0.10±0.01	0.29±0.05	0.04±0.00	-	-	-	-	0.01±0.00	-	0.03±0.00	-
	Total	3.18±0.06	11.43±0.14	5.27±0.13	10.30±0.09	3.17±0.10	0.68±0.01	0.31±0.02	0.31±0.02	0.06±0.00	1.33±0.02	0.49±0.01	3.05±0.07	1.43±0.06
		**Ferulic acid derivatives[b], μg mL^{-1}**												
FA1	Ferulic acid -O-glucoside I	-	-	3.80±0.52	-	-	-	-	-	-	-	-	-	-
FA2	Ferulic acid -O-glucoside II	2.96±0.35	11.16±0.85	8.79±0.64	10.64±1.23	4.39±1.05	0.26±0.03	0.08±0.01	0.34±0.05	0.13±0.01	0.30±0.02	0.30±0.04	-	0.35±0.01
FA3	Ferulic acid -O-glucoside III	0.85±0.13	2.92±0.32	2.14±0.35	2.89±0.65	0.71±0.15	0.03±0.00	-	0.43±0.07	-	-	0.23±0.02	-	-
	Total	3.80±0.16	14.08±0.37	14.74±0.15	13.54±0.41	5.11±0.64	0.29±0.02	0.08±0.01	0.77±0.01	0.13±0.01	0.30±0.02	0.53±0.01	-	0.35±0.01
		**Coumarin derivatives[c], μg mL^{-1}**												
COU1	Skimmin	0.86±0.17	3.42±0.24	2.24±-0.13	3.34±0.33	0.79±0.14	-	-	-	-	-	-	-	-
COU2	Umbelliferone	1.54±0.23	4.42±0.32	0.54±0.06	1.49±0.10	0.35±0.06	0.92±0.19	0.28±0.04	0.55±0.16	0.51±0.07	1.87±0.24	0.74±0.15	6.53±0.44	2.35±0.25
COU3	Herniarin	16.59±2.37	11.35±0.57	5.21±0.64	1.62±0.18	6.63±0.58	28.36±1.27	11.92±0.88	14.75±0.97	6.12±0.39	34.94±1.83	21.56±1.34	43.28±1.51	35.85±1.13
COU4	(3'x,5'a,9'x,10'b)-O-(3-Hydroxy-6-oxo-7-drimen-11-yl)umbelliferone	0.77±0.12	3.06±0.22	1.44±0.26	2.16±0.23	0.35±0.08	-	-	-	-	0.10±0.00	-	0.20±0.01	-
	Total	19.76±1.03	22.25±0.16	9.42±0.26	8.62±0.10	8.12±0.25	29.27±0.76	12.20±0.49	15.31±0.57	6.63±0.23	36.91±0.99	22.29±0.84	50.01±0.77	38.20±0.62
		**Apigenin derivatives[d], μg mL^{-1}**												
API1	Apigenin 7-O-glucoside	1.88±0.16	6.87±0.33	7.48±0.54	6.57±0.52	3.78±0.19	0.66±0.12	-	-	-	-	0.33±0.03	-	-
API2	Apigenin-7-O-(6''-acetyl-gulcoside)	1.21±0.13	4.41±0.25	3.75±0.46	3.46±0.49	1.26±0.22	0.21±0.01	0.21±0.02	-	-	-	0.10±0.00	-	-
API3	Apigenin-7-O-(4''-acetyl-gulcoside)	0.57±0.10	2.02±0.17	1.82±0.83	1.83±0.22	0.65±0.05	0.11±0.01	0.15±0.01	-	-	-	0.06±0.00	-	-
API4	Apigenin-7-O-(6''-malonyl,4''-acetyl-gulcoside)	1.24±0.11	4.17±0.33	4.79±0.67	3.42±0.31	2.18±0.16	0.29±0.08	0.52±0.09	-	-	0.04±0.00	0.15±0.01	0.05±0.00	0.05±0.00
API5	Apigenin-7-O-(4''-malonyl,6''-acetyl-gulcoside)	6.05±0.37	23.86±1.02	2.53±0.23	1.80±0.17	0.90±0.12	0.14±0.01	0.22±0.01	-	-	0.01±0.00	0.07±0.01	0.02±0.00	0.02±0.00
API6	Apigenin	1.82±0.42	7.00±0.66	5.19±0.91	3.64±0.15	1.12±0.28	0.29±0.06	-	-	0.11±0.01	0.24±0.02	0.15±0.01	0.31±0.03	0.26±0.02
	Total	12.78±0.14	48.32±0.32	25.55±0.25	20.73±0.16	9.89±0.08	1.70±0.05	1.09±0.04	-	0.11±0.01	0.29±0.01	0.85±0.01	0.37±0.02	0.32±0.01

[a]Leucine equivalents; [b]Ferulic acid equivalents; [c]Coumarin equivalents; [d]Apigenin equivalents; a blank indicates that the compound has not been detected.
